# Acknowledgment to the Reviewers of *Biomedicines* in 2022

**DOI:** 10.3390/biomedicines11020266

**Published:** 2023-01-19

**Authors:** Biomedicines Editorial Office

**Affiliations:** MDPI AG, St. Alban-Anlage 66, 4052 Basel, Switzerland

High-quality academic publishing is built on rigorous peer review. *Biomedicines* was able to uphold its high standards for published papers due to the outstanding efforts of our reviewers. Thanks to the efforts of our reviewers in 2022, the median time to first decision was 17 days and the median time to publication was 38 days. Regardless of whether the articles they examined were ultimately published, the editors would like to express their appreciation and thank the following reviewers for the time and dedication that they have shown *Biomedicines*:
A. N. M. Mamun-Or-RashidKlaudia BanachAbbas RahdarKlaudia SztolsztenerAbdelkarim AbousalhamKlaudija ViškovićAbdelouahed KhalilKlaus JungAbdollah AllahverdiKlaus-Dieter SchlüterAbdul MajeedKlemen BohincAbdullah BayramKo OkumuraAbdullah Bin ShamsKohei OkuyamaAbdullah ShaitoKohei TakahashiAbedalrhman AlkhateebKohei UchimuraAbhijit AithalKohji FukunagaAbhik SahaKoichi KawadaAbhilash SamykuttyKoichi MatsuoAbhishek GuptaKoji OtaniAbid UllahKok Hian TanAbigail SuwalaKok Min SeowAbubakr H. MossaKomal VigAdam GolabKomuraiah MyakalaAdam M. KayeKondababu KurakulaAdam ReichKondareddy CherukulaAdam W. WhisnantKongju ZhuAdam WittekKonrad A. SzychowskiAdebayo Olatunbosun SojobiKonrad RudnickiAdele StewartKonstantin A. KrychtiukAdeline DivouxKonstantin BelosludtsevAdina Turcu-StiolicaKonstantin N. YaryginAdnan KhanKonstantin V. SlavinAdolfo Gabriele MauroKonstantinos DimasAdrian ArendowskiKonstantinos KamposiorasAdrian AugustyniakKonstantinos MengrelisAdrian NeaguKonstantinos VlachosAdriana CalderaroKorhan BuyukturkogluAdriana ChilinKornelia Kuźnik-TrochaAdriana GeorgescuKoteswara Yerra RaoAdriana GrigorașKoustav GangulyAdriana M. CalvoKoya OzawaAdriana VinhasKrešimir RotimAdrian-Bogdan ȚiguKrinio GiannikouAdriano MollicaKripa ShankarAdriano PiattelliKrishna K. SharmaAdriano SilvaKrishna KantAdrija HajraKrishnendu PalAfra WohlschlägerKristian T. SchafernakAgata GóźdźKristina EndresAgata MichalakKristina Pogrmic-MajkicAgata Poniewierska-BaranKristine GriffettAgata SebastianKristine KrajnakAgata StanekKristine M. KrajnakAgieshkumar Balakrishna PillaiKristóf Endre KárolyAgnes KlarKristoffel R. DumonAgnes SchröderKrisztina PohóczkyÁgnes TelbiszKrupa NavalkarAgnese De MarioKrystelle Nganou-MakamdopAgnese PoKrystyna Mazan-MamczarzAgnese SbrolliniKrystyna PawlakAgnieszka Bojarska-JunakKrystyna Pierzchala-KoziecAgnieszka BossowskaKrzysztof BilminAgnieszka Jagiello-GruszfeldKrzysztof CzamaraAgnieszka KosowskaKrzysztof GomułkaAgnieszka MagryśKrzysztof GoryńskiAgnieszka Matera-WitkiewiczKrzysztof MuchaAgnieszka Owczarczyk-SaczonekKrzysztof SzyfterAgnieszka SliwinskaKrzysztof TadyszakAgnieszka ŚmieszekKrzysztof TupikowskiAgnieszka StawarskaKsenija DurgoAgnieszka SzewczykKuldeep K. BansalAgnieszka WąsikKumánovics GáborAgnieszka WnukKumar Chandan SrivastavaAgnieszka ZabłockaKumar GanesanAgnieszka ZagórskaKun WangAgnieszka ZawiejskaKun YangAgnieszka Zelek-MolikKundlik GadhaveAgustin Gonzalez-VicenteKunihiko HiramatsuAhmad SaltiKunjan R. DaveAhmed Abdal DayemKun-Ling TsaiAhmed AbdelhakKuo-Chung LanAhmed BakillahKusheng WuAhmed MostafaKuthati YaswanthAhmet AyazKwang Mook JungAida PetcaKwang Suk LimAinhoa Iglesias-AraKwong-Hang TamAinhoa Plaza-ZabalaKyeong Sook ChoiAiqing LiKyle BurghardtAi-Ru HsiehKylie D. RockAishwarya GurumurthyKyoung-Min ParkAishwarya IyerKyung Pyo KangAiti VizziniKyung Rae KoAitor LarrañagaKyunghoon MinAiva PlotnieceKyungsik EomAjay DixitKyung-Yil LeeAjay PalKyu-Sang ParkAjay Vikram SinghKyuseok KimAjit RoyLacramioara PopaAjmer Singh GrewalLaith AlzubaidAkanksha SharmaLang TranAkanksha VermaLara BaticicAkhtar AliLara BielerAkihiko HiyamaLara GibelliniAkihiko KatayamaLarisa AnghelAkihiko NishikimiLarisa LitvinovaAkihiko YamamotoLarisa RyskalinAkihiro YoshidaLarisa Viktorovna SuturinaAkihisa TakahashiLarisa VolovaAkihito TsubotaLars HeggelundAkinobu OtaLars HelbigAkinori SatoLars KaestnerAkira YamauchiLars P. KamolzAkito NakagawaLászló HéjaAkitoshi SanoLászló JanovákAkshaya BhagavathulaLászló MangelAlaa Abd-ElsayedLászló SzabóAlaa GhaliLászló VécseiAlaguvel ValliammaiLatefa DardasAlain AttarLatifa BouissaneAlan G. GoodmanLaura AudìAlan PalmerLaura Carolina Zanetti-DominguesAlan RemaleyLaura CrisponiAlana Aragón-HerreraLaura FranzaAlarico ArianiLaura Gheuca-SolovastruAlban Fouasson-ChaillouxLaura M. FragoAlbert PoaterLaura MarchettiAlbert StewartLaura MauriAlberto Comesaña-CamposLaura RigonAlberto FerrareseLaura VitielloAlberto Lo GulloLaura XicotaAlberto MellaLauren DoumaAlberto Rodríguez-ArchillaLaurens W. J. BosmanAlberto SignoreLaurent L’hommeAlbino BacollaLaurent MagyAlbino CarrizzoLaurent MetzingerAlbino EccherLaurent PeyrodieAldona SiennickaLaurentiu M. PopAlejandra Garcia-GascaLaurie M. ElitAlejandro A. SchäfferLavinia Cosmina ArdeleanAlejandro BugarinLawrence I. GrossmanAlejandro Schcolnik-CabreraLayla PiresAleksander MendykLe Nguyen Quoc KhanhAleksandr A. RubelLeela Raghava Jaidev ChakkaAleksandra BukhovetsLeena Hilakivi-ClarkeAleksandra FucicLeena P. BharathAleksandra GlogowskaLei ChangAleksandra JungLei FangAleksandra Karczmarska-WódzkaLei HeAleksandra KlimczakLei LiAleksandra M. BondžićLei NieAleksandra Majchrzak-CelińskaLei WangAleksandra Piechota-PolanczykLena Nilsson-WikmarAleksandra Rajewska-RagerLene Karine VestbyAleksandra RogowskaLenka MichalcovaAleksandra S. KristoLeonardo BencivengaAleksandra Sałagacka-KubiakLeonardo PaganiAleksandra SzopaLeonid A. KaluzhskiyAleksandra Wisłowska-StanekLeonor M. MeiselAleksei TikhonovLeopold EckhartAleksey Michailovich ChaulinLeopoldo González-BrusiAleksey ZaitsevLeslie CaronAleš BlincLetizia GiampietroAlessandra BigiLewis Mehl-MadronaAlessandra ColciagoLeyre ZubiriAlessandra CrisàLi FuAlessandra De MatteisLi LinAlessandra FerramoscaLia Mara DiţuAlessandra MarchesiLia PulsatelliAlessandra PalladiniLiana FattoreAlessandra PierangeliLiang-Yo YangAlessandra QuartaLibero VitielloAlessandra SinopoliLiborija Lugović-MihićAlessandra ValerioLiborio StuppiaAlessandro AntonelliLi-Chen ChenAlessandro BuonomoLichung ChiuAlessandro CeliLicun WuAlessandro ComandoneLidia GaffkeAlessandro D’AmuriLidia Hanna MarkiewiczAlessandro de SireLidia Szulc-DąbrowskaAlessandro DelitalaLidija GradišnikAlessandro FassinaLieven VlaminckAlessandro GrinzatoLige TongguAlessandro MalobertiLigen YuAlessandro MassaroLigia PetricaAlessandro MeduriLihua ChenAlessandro OttaianoLi-Jen LeeAlessandro PoggiLikang ChinAlessandro RizzoLilach SoreqAlessandro RolfoLiliang JinAlessandro RussoLin XiAlessandro ScalaLinbo ZhaoAlessia CarboniLinda J. Larson-PriorAlessia FilipponeLing WangAlessia LodiLing YinAlessia RosiLing-Wen DingAlessio ArdizzoneLinto ThomasAlessio BocediLipika ChablaniAlessio ContiLiqiang WangAlessio GnerucciLiquan HuangAlex Al KhouryLiron RozenkrantzAlex M. ValmLisa M. ArendtAlexander A. ZhgunLisa M. GallagherAlexander BrillLisa McFetridgeAlexander BunevLiubov GorbachevaAlexander E. BerezinLiudmyla StorozhukAlexander GasparskiLivia Di RenzoAlexander J. MentzerLivia LenziniAlexander KabakovLiviu SteierAlexander RingLivius V. D’UscioAlexander RueschLiwei YangAlexander ShtilLixia HeAlexander TamalunasLiya XuAlexander TyshkovskiyLi-Yen ShiuAlexander V. MaltsevLjiljana Gojković-BukaricaAlexander V. ProninLjudmila StojanovichAlexandra BocsikLogan K. TownsendAlexandra BurluiLoic LemonnierAlexandra Cristina MunteanuLoka Raghu Kumar PenkeAlexandra FoscolouLondon L. P. J. OoiAlexandra GouveiaLong Chiau MingAlexandra GyongyosiLong-Sheng LuAlexandra KhlyustovaLoredan Stefan NiculescuAlexandra Ripszky TotanLoredana MarcuAlexandra TsidulkoLoredana UrsoAlexandre BuffetLoreé C. HellerAlexandre González-RodríguezLorelei Irina BrasoveanuAlexandre QuintasLorena Elena MelitAlexandrina BurlacuLorena GurrieriAlexandrina Ferreira MendesLorena UrbanelliAlexandru BurlacuLorenzo NeviAlexandru PopaLorenzo PaceAlexandru UliciLoris PironiAlexei ChukhlovinLourdes Cortes-DericksAlexey BoykoLovorka BrajkovićAlexey ChubarovLu CaiAlexey ChurovLu LiAlexey DeykinLu WangAlexey FayzullinLubomir SkladanyAlexey KovalLuca AmbrosioAlexey SarapultsevLuca Di GianfrancescoAlexey SokolovLuca FalzoneAlexios Vlamis-GardikasLuca FilippiAlexis TalbotLuca GallelliAlfonso Martínez-NovaLuca GiacomelliAlfredo BellonLuca Giovanni LocatelloAlfredo Cabrera OreficeLuca PaciniAlfredo CaturanoLuca Proietti De SantisAlfredo PapaLuca TestarelliAlfredo PulvirentiLucas Taoro-GonzálezAlfredo Saavedra-MolinaLuci DusseAli AhmadLucia BrodosiAli BoolaniLucia ČernákováAli KaissiLucía Fernández CasanovaAli MahtaLucia MorbidelliAli R. JazirehiLucia RoccoAli ZarrabiLuciana BordinAlice BonomiLuciana Pereira RangelAlice LuddiLucille LumleyAlice VerticchioLucinda J. BessaAlice Verticchio VercellinLucinda V. ReisAlicia ChenowethLucio Della GuardiaAlicja KluczykLucio Díaz-FloresAlin CiobicăLucjusz ZaprutkoAlina-Andreea ZimtaLucy NorrisAline Yen Ling WangLucyna Pomierny-ChamiołoAlisa SokolovskayaLudek SefcAlisha PrasadLudek SlavikAlison E. Fox-RobichaudLudmila KazdovaAllison Ashley-KochLudmila PaškováAlpa A. TrivediLudovica NucciAlphus D. WilsonLudovico MessineoAlpo VuorioLuigi Alberto PiniAlthaf ShaikLuigi BennardoAlvaro LaraLuigi CristianoÁlvaro Santana-GarridoLuigi Della CorteAlvise SernicolaLuigi RosatiAmalia MesarosLuigi SansoneAmalia StefaniuLuigi SchipsAmanda IglesiasLuigi TritapepeAmanda M. NelsonLuigi VitaglianoAmanda WallerLuigia BruglieraAmanda WasylishenLuigi-alberto PiniAmani Yousef OwdaLuís GalesAmany MohamedLuis I. TerrazasAma-Tawiah EssilfieLuis J. RoyoAmbrogio P. LonderoLuis Jesús Villarreal-GómezAmeeduzzafar ZafarLuis Manuel TeranAmélie GuihotLuis Marinas-PardoAmer AhmedLuís Pinto Da SilvaAmerigo GiudiceLuís RatoAmi RavalLuis RodrigoAmir Amiri-YektaLuisa RicciardiAmir Mohammad MalvandiLuiza Ioana GăinăAmir ShemiraniLukas D. LandeggerAmirbabak Sioofy-KhojineLukas LacinaAmit K. MaitiŁukasz A. PoniatowskiAmit KumarŁukasz DobrekAmit TripathiŁukasz M. MilanowskiAmitava MukherjeeLukasz MalekAmiya PatraŁukasz SobottaAmjad KhanŁukasz SzeleszczukAmmad Ahmad FarooqiŁukasz SzymańskiAmnon HorovitzŁukasz WięcławAmol KulkarniŁukasz ZapałaAmosy E. M’KomaLuke ParkitnyAmr AminLulu CaiAmra AdroviçLuminiţa MoraruAmrendra K. AjayLuminita StanciuAmrita SarkarLu-Ting KuoAmy MillenLutz KonradAna Babic PerhocLydia Giménez-LlortAna BanitoLydia Min-Ying SuAna Bela Sarmento-RibeiroLynn M. PezzaniteAna Blas-GarcíaLynn PulliamAna C. PinhoLyudmila Bel’skayaAna Carolina GonçalvesLyudmila DimitrovaAna CaruntuLyudmila RachekAna Catarina TorresM. R. MozafariAna CicvaricMaarit TiirikainenAna Colette MaurícioMaciej DaniszewskiAna Elena Rodríguez-RodríguezMaciej JarzębskiAna Fernández-AraqueMaciej JedlińskiAna I. ArrobaMaciej PrzybyłekAna Isabel FloresMaciej SikoraAna Isabel Fraguas-SánchezMaciej WnukAna M. SahagunMaddalena FratelliAna Margarida PereiraMaddalena NapolitanoAna P. SantosMadelyn A. GillentineAna PlácidoMadelyn GillentineAna Podolski-RenićMadhavan NampoothiriAna Raquel Santa-MariaMadhumita ChatterjeeAna Rita FerreiraMagda R. HamczykAna Rita Xavier De Jesus GameiroMagdalena BartnikAna Rosa Rincón-SánchezMagdalena Chottova DvorakovaAna TadijanMagdalena GórnickaAna TomićMagdalena GrillAna-Bárbara García-GarcíaMagdalena HurkaczAnabela Baptista Pereira PaulaMagdalena Krajewska-WłodarczykAnaïs Pitto-BarryMagdalena KrbotAnamaria JurcauMagdalena OgłuszkaAnamaria SavuMagdalena RauschAna-Maria UdreaMagdalena StobieckaAnand SinghMagdalene J. SeilerAnanta Prasad ArukhaMahendran SekarAnas ShamsiMahesh AttimaradAnastasia KotanidouMahima SinghAnastasiia PetushkovaMahmoud BalbaaAnastasiya SolovievaMahmoud ShehataAnca ColitaMaja Jazvinšćak JembrekAnca DinischiotuMaja MaticAnca GafencuMaja MatulićAnca Giorgiana GrigorašMajdy IdreesAnca Maria PanaitescuMajid Mufaqam Syed-AbdulAnca Niculina CadinoiuMakoto HasegawaAnca Oana DoceaMakoto MakishimaAnca Roxana PetroviciMalgorzata BurekAnca UdristoiuMałgorzata KajtaAnca UngurianuMałgorzata KapralAncel JulienMałgorzata Kotula-BalakAn-Chen ChangMałgorzata KujawskaAnder Vergara AranaMalgorzata Maria JerzakAnders MellemgaardMałgorzata PrzybyłoAndleeb KhanMałgorzata SzczukoAndrás BallaMałgorzata Zalewska-AdamiecAndras BanvolgyiMalik AydinAndras BikovMallikharjuna KoriviAndraẑ DovnikManabu NiimiAndre HussMandy BeyerAndré M. N. SilvaManel Ben HammoudaAndré M. Van RijMangesh HadeAndré ObenausManickam AshokkumarAndré VisManohar KodavatiAndrea AngelettiManoj Kumar TembhreAndrea BalliniMansour SobehAndrea BeccioliniManu PrasadAndrea ButeraManuel A. ValdebranAndrea CaporaleManuel Freire-GarabalAndrea De PieriManuel MuroAndrea FariniManuel PestanaAndrea GelemanovićManuel Rosa-GarridoAndrea GianniniManuel Sanchez-MartinAndrea Gonzalez-MontoroManuela LeriAndrea Hulina-TomaškovićManuela MontanaroAndrea HuwilerManuela Sofia Rodrigues MoratoAndrea LapucciMao-Meng TiaoAndrea LatiniMara CarsoteAndrea M. P. RomaniMāra PilmaneAndrea MagrìMarc André LécuyerAndrea ManciniMarc DeanAndrea MariMarc FlajoletAndrea MohrMarc MarescaAndrea MulliriMarc ParmentierAndrea OrsiMarc PoirotAndrea Pereira RegnerMarcel AdlerAndrea PerrelliMarcel BonayAndrea PiccinMarcela Bermúdez EcheverryAndrea RaymondMarcella RealeAndrea RoccuzzoMarcella TazzariAndrea RomaniMarcello IasielloAndrea S. MelaniMarcello PintiAndrea SansoneMarcello TagliaviaAndrea SantarelliMárcia BarrosAndrea ScharfMarciane MilanskiAndrea SistiMarcin ArciszewskiAndrea StoccoroMarcin BasiakAndrea SvoradováMarcin DelijewskiAndrea TittarelliMarcin JaneckiAndrea TrevisanMarcin RóżalskiAndrea VenerandoMarcin RudzkiAndreadi AikateriniMarcin SiwekAndreas FischerMarcin SochalAndreas IhleMarcin WekwejtAndreas J. RiethMarco BattistaAndreas KoupparisMarco Bauzá ThorbrüggeAndreas KriegMarco BoAndreas KurtzMarco BucciAndreas NeuederMarco C. CavacoAndreas OuranidisMarco CarmignaniAndreas PetryMarco ChinoAndreas von KnethenMarco CicconeAndreea IacobMarco G. AlvesAndreea Lili BărbulescuMarco GalluzzoAndreea-Catarina PopescuMarco InvernizziAndrei Gheorghe MotocMarco La VerdeAndrei SurguchovMarco MainardiAndrei Vasile NastutaMarco Matucci CerinicAndreia Z. ChignaliaMarco PalmaAndrej MarkotaMarco PortelliAndrés Alonso Agudelo-SuárezMarco RohdeAndres López-ContrerasMarco SalvatoreAndrew CovelerMarco ScarselliAndrew CsordasMarco SetteAndrew D. FrugéMarco TallaricoAndrew G. VolkMarco TatulloAndrew GetahunMarco ZeddaAndrew J. WoodMarcos Arturo Martínez-BanaclochaAndrew MallettMarcos Soto-HernándezAndrew NorrisMarcus DymondAndrey AbramovMarcus KrügerAndrey ElchaninovMarcus LancéAndrey GlotovMarcus Schmitt-EgenolfAndrey KozlovMarcus Tullius ScottiAndrey SudarikovMarek DrozdzikAndrzej CzyrskiMarek LangnerAndrzej KoniecznyMarek LapkaAndrzej KubiakMarek MuriasAndrzej LangeMarek SkrzypskiAndrzej PawlikMarek WesołowskiAndrzej PolanczykMargarida Duarte AraújoAndrzej PomianowskiMargarita A. SazonovaAndrzej PrzybylskiMargarita Jimenez-PalomaresAndrzej SlominskiMargarita MaurerAndrzej WędrychowiczMargarita TenopoulouAndrzej Za̧bekMargaux Jenna KanisAndy LauMargherita EufemiAnelia HorvathMargherita IzziAnella SavianoMargherita NeriAneta Ostróżka-CieślikMargherita-Gabriella de BiasiAneta PopovaMari ChiritoiuAneta ŚcieżyńskaMaria A. BaturovaAnette Gjörloff WingrenMaria A. VulfAngel AlvarezMaria AmbrosioAngel Buendía-RomeroMaria Angeles RojoAngel GalabovMaria Angelica MiglinoAngel M. CuestaMaria BovaÁngel Rodríguez-VillodresMaria C. AlonsoAngela BriegerMaría Camacho EncinaAngela Dalia RicciMaria Chiara GattoAngela De SimoneMaria CorreiaAngela Di SommaMaria Cristina TanziAngela DziedzicMaria D. Giron-GonzalezAngela FleischhackerMaria da Graça Costa MiguelAngela IvaskMaria De GrandisAngela PucciMaria de las Mercedes Garcia-GilAngela RizziMaría Del Carmen Carcelén-FraileAngela StaicuMaría Dolores Llobat BordesAngelica LoskogMaria Dulce EstêvãoAngeliki AngelidiMaria E. MycielskaAngélique SourMaria Elena MelicaAngelo RegaMaría Esperanza CerdánAngelo RuggieroMaría Eugenia Jaramillo FloresAngelos SikalidisMaría GalánAngom Ramcharan SinghMaria GavriilakiAniello AlfieriMaria Giovanna ScioliAniello FrancescoMaria Gomes Da SilvaAniello MaieseMaria Grazia MancinoAnil SinghMaria Grazia MolaAnil TharappelMaría Janeth Rodríguez-RoqueAnis HannaMaría Jesús Núñez-IglesiasAnişoara CîmpeanMaria Joana PintoAnita Lyssek-BorónMaría José Bautista-LlamasAnita QuintanaMaría José García-PolaAnja Kathrin JaehneMaría José Ruiz-MagañaAnja ZeigererMaria Lauda TomasiAnjali KusumbeMaria Lisa GaravagliaAnju KumariMaria Luiza S. MelloAnke LangenfeldMaría M. Morales Suárez-VarelaAnkit SanejaMaria MartellAnkit SrivastavaMaria MatuzAnkita PunethaMaria MoschoviAnkita SrivastavaMaria NikolovaAnkona BanerjeeMaria NotarnicolaAnn SaadaMaría Orosia Lucha-LópezAnna A. KashevarovaMaria Paola BertuccioAnna Arnal-GómezMaria PaparoupaAnna AtlanteMaria PassanteAnna B. VolnovaMaria Pia FelliAnna BaraniakMaria Pia Foschino BarbaroAnna Bednarska-CzerwińskaMaria R. BonsignoreAnna BenrickMaria Rita BiancoAnna BizońMaria Rita CiteroniAnna CalarcoMaria RomoAnna CampanatiMaria Rosaria De MiglioAnna ChoromanskaMaria Salomé GomesAnna Cleta CroceMaria SamaraAnna Di MatteoMária SzekeresAnna DrabczykMaria TourakiAnna ErmundMaria TrovatoAnna FialováMaría U. Moreno ZulateguiAnna FilipekMaria V. TurovskayaAnna GalickaMaría Val Toledo LoboAnna GiammancoMaria VenihakiAnna Jamroz-WisniewskaMaria Vincenza CataniaAnna Kabłak-ZiembickaMaría Virumbrales-MuñozAnna Kurek-GóreckaMaria ViscomiAnna L. GharibyanMaria Vittoria CubellisAnna LaskowskaMaria-Alexandra MartuAnna LisowskaMariadoss Arokia Vijaya AnandAnna Maria NuzzoMaria-Jimena Mucino-BermejoAnna Merecz-SadowskaMariana Carmen ChifiriucAnna MichnoMariana FerreiraAnna N. BerlinaMariana FloriaAnna NegroniMariana FragosoAnna OgrodowczykMariana Pavel-TanasaAnna OniszczukMariana SantosAnna Paradowska-StolarzMariana SponchiadoAnna PellatMariana Varna-PannerecAnna PetruczynikMariangela ManciniAnna Pokryszko-DraganMariann HarangiAnna SerefkoMarianna ButtarelliAnna StarzyńskaMarianna ChristodoulouAnna StefanskaMarianna MazzaAnna Szóstek-MioduchowskaMarianna RanieriAnna Tabęcka-ŁonczyńskaMarianne BerwickAnna Wai San CheangMariano Francesco CaratozzoloAnna WojciechowskaMariantonietta Di StefanoAnna Y. AksenovaMaribella DomenechAnna Zalewska-JanowskaMaricica PacurariAnna-Leena SirénMarie CarlsonAnnalisa CappellaMarie Cécile NassogneAnnalisa ChiavaroliMarie Saghaeian JaziAnnamaria FranzèMarie Thérèse VanierAnnamaria SandomenicoMarie WalshAnnapina RussoMarie-Louise ElkjaerAnnarita NappiMarieta I. TomaAnne CaignardMarija Pljesa-ErcegovacAnne JarryMarijn SpeeckaertAnne Régnier-VigourouxMarike W. Van GisbergenAnnegret Pelchen-MatthewsMariko OkamotoAnne-Sophie WoznyMarilena GaldieroAnnet KiraboMarilena GregoriniAnnette GrahamMarin MicutzAnnibale PucaMarina AunapuuAnnunziata MauroMarina Consuelo VitaleAnnunziata NuscaMarina JovanovićAnny Claude LuissintMarina KhodanovichAnon SrikiatkhachornMarina LeitmanAnoop ArunagiriMarina Marina ElliAnoop RawatMarina MencingerAntal RockenbauerMarina P. IsaevaAnthony J. BaucumMarina PiscopoAnthony KondrackiMarina QuartuAnthony William ColemanMarina Ruxandra OteleaAnton I. KorbutMarina SagudAnton M. ManakhovMarina SalaAnton P. TyurinMarina Villanueva-PazAnton R. KiselevMarinella CocoAnton TyurinMarino VilovićAnton V. KiselevMario AbateAntonella CaccamoMario CioceAntonella D’AgostinoMario G. MirisolaAntonella FerranteMario GanauAntonella FontanaMario Vincenzo RussoAntonella GozziniMariola JaniszewskaAntonella LisiMarios G. KrokidisAntonella OliveroMarios SpanakisAntonella PaladinoMarisa Gabriela RepettoAntonella RomaniniMarisa VarrentiAntonella RosiMarish I. F. J. OerlemansAntonella SmeriglioMarius L. CrainaAntoni BenitoMarius RademakerAntonia FeolaMariusz DabrowskiAntonia PiazzesiMariusz HartmanAntonia SiogaMariusz KlenckiAntonieta Guerrero-PlataMariusz MojzychAntonietta LiottiMariusz SikoraAntonija TadinMarjan MohammadiAntonino TuttolomondoMarjeta TerčeljAntonio BiondiMarjolaine WillemsAntonio ColantuoniMarjorie Cornejo PontelliAntonio Díaz-QuintanaMarjorie JonesAntonio FacciorussoMark C. HowellAntonio G. SolimandoMárk FráterAntonio GaballoMark M. WalshAntonio GidaroMark PlazierAntónio GinjeiraMark WilliamsAntonio GiovinoMarko LucijanićAntonio Junior LepeddaMarko PopovicAntonio LeoMarkus FendtAntonio Martinez-LopezMarkus GlaßAntonio MarzolaMarkus M. HoffmannAntonio MolinoMarkus MandlAntonio OrlacchioMarkus WolfienAntonio Simone LaganàMarlena ZyśkAntonio TrovatoMarsha PierceAntonio TursiMarta AlonsoAntonis SakellariosMarta BalaskoAntonius M. J. VanDongenMarta Camprubí-RimblasAnuhya KommalapatiMarta F. EstradaAnukul T. ShenoyMarta GaburjákováAnupam DhasmanaMarta GomarascaAnupam MukherjeeMarta GrabowskaAnuraag BoddupalliMarta Joanna Szandruk-BenderAo ZhangMarta KepinskaApinun KanpiengjaiMarta MasztalewiczApostolos PapachristosMarta NardiniApostolos ZaravinosMarta Regina Cezar-VazArabinda DasMarta S. CarvalhoArati SridharanMárta SárközyArchana MishraMarta Seco-CerveraArghya RayMarta SzachniukArianna CasciatiMarta TomczykAriel FurerMarta VerneroArif KhanMarta WłodarczykArif LuqmanMartha SkerrettArijit SenguptaMartin BakhtArindam PramanikMartin Bjørn StausholmAris TsalouchosMartin GausterArkadij SobolevMartin J. HoogduijnArkadiusz P. MatwijczukMartin M. MortazaviArmando CardosoMartin MistrikArmando CaseiroMartín Pérez-SantosArnab GhoshMartin PestaArnab SenMartin SztachoArnaud MailleuxMartin TeraaArnaud ScherberichMartin VlkArrigo CiceroMartin WasserArsalan HaqqaniMartina KroppArseniy LobovMartino L. Di SalvoArthi ShridharMartynas SimanaviciusArtur PałaszMarvin Antonio Soriano-UrsúaArtur SlomkaMary Cano-SarabiaAruna SharmaMaryam ArdalanArvind NegiMaryam FarooquiArvind PandeyMarzena LaskowskaArzu BakirtasMasaaki KonishiAsad JanMasabumi ShibuyaAsada LeelahavanichkulMasafumi MurataniAshenafi F. BeyiMasahiko YamaguchiAshfaqul HoqueMasahiro AkiyamaAshish JainMasahiro ImamuraAshok AspatwarMasahito YamagataAshok IyaswamyMasaki OkamotoAshok PatowaryMasamichi IkawaAshraf Yusuf RangrezMasanori KatakuraAshu JohriMasanori MurakamiAshutosh P. DubeyMasao KodaAshutosh PandeyMasao OtaAsker KhapchaevMasaru MiyazakiAssunta SellittoMasaru TanakaAsta JudžentienėMasashi ToyodaAsterios (Stergios) PispasMasataka KikuyamaAstrid HammerMasato YanagiAstrid M. LiedertMasaya TachibanaAswin L. MenkeMasayuki ItohAtefeh ZarepourMasood Alam KhanAthanasios AlexandrisMasoud KeikhaAthanasios SalifoglouMassimiliano AmmirabileAthanassios TselebisMassimiliano CadamuroAtsuki NaraMassimiliano CaiazzoAtsushi MasamuneMassimiliano ChettaAtsushi NagaiMassimiliano MangoneAtsushi TanakaMassimiliano TognoliniAttila FarsangMassimiliano VerouxAttila FrigyMassimo BergerAttila TothMassimo CapocciaAtul DaiwileMassimo ChelloAudrey CrasMassimo ConeseAudrey GuenicheMassimo CorsaliniAudrey VarinMassimo LibraAugust WrotekMassimo LorussoAugustin M. OfiteruMassimo MaccagnoAurel Popa-WagnerMateusz ŁucAurora ArghirMateusz PierógAurora SavinoMathew AlexanderAvathamsa AthirasalaMats ErikssonAvinash GothwalMatteo Alessio ChiappediAvinash WaghrayMatteo BaucknehtAvinoam NevlerMatteo Bruno LodiAxel PagenstecherMatteo De PastenaAyataka FujimotoMatteo FermiAyla YagdiranMatteo FerroAyman J. OweidaMatteo GiovarelliAyman MahmoudMatteo GiuliettiAyokanmi OreMatteo Luigi Giuseppe LeoniAyrat ZiganshinMatteo MarcacciAysegül AksanMatteo MegnaAyumi YoshizakiMatteo SambucciAyyanar SivananthamMatthew F. CusickAzad R. BhuiyanMatthew G. K. BeneschAzadeh AsefnejadMatthew Q. MillerAzhar IlyasMatthew R. PincusAzizur RahmanMatthew Stuart McKinneyAzzurra StefanucciMatthias ElsnerBabett RamsauerMatthias Hardtke-WolenskiBach Quang LeMatthias KarlBachir BenarbaMatthias LauthBahia HakikiMatthias U. NollertBahil GhanimMatti JauhiainenBahriye AktasMattia Di PaoloBai Chuang ShyuMattia Falchetto OstiBal Krishna ChaubeMatus DurdikBalachandar VellingiriMatus SykoraBalaji PanchapakesanMaulik VyasBalázs MayerMaurice GiroudBalazs SarkadiMaurizio CrestaniBálint NagyMaurizio MarchesiniBálint TamaskovicsMaurizio MolinariBallambhattu Vishnu BhatMaurizio PascadopoliBalupillai AgilanMaurizio RomagnuoloBanhi BiswasMaurizio StellaBaoye HeMauro ColucciaBarbara CuomoMauro GiuffrèBarbara GrzechocińskaMauro MontalbanoBarbara KaltschmidtMaw-Sheng LeeBarbara M. WirostkoMaxim SorokinBarbara MavroidiMaxim V. BerezovskiBarbara MognettiMaxime PichonBarbara PergolizziMaximiliano Tamae KakazuBarbara RuaroMaya DymovaBarbara Ruszkowska-CiastekMayank ChoubeyBarbara SchnierleMayumi NishiBarbara SpanòMayur DokeBarbara SteccaMd Afjalus SirajBarbara ToffoliMd MohiuddinBarbara VizioMd. Imtaiyaz HassanBarbara ZdzisińskaMehdi MehraliBarbora KaliňákováMehdi MontazerBart De GeestMehdi SyedBartlomiej BarczynskiMehrnoosh Saghizadeh GhiamBartłomiej PerekMei ZhaoBartosz ChmielowskiMeiyan WangBartosz ForoncewiczMeiyun FanBartosz MalkiewiczMeizhu BaiBartosz SzmydMejía CarmenBaruh PolisMelánia BabincováBashayer Al MubarakMelinda HerbathBasheer MannasahebMelinda SzilágyiBashoo NaziruddinMelissa García-CaballeroBaskaran InbarajMelvin HaydenBassam AbomoelakMenachem NahirBassant BarakatMeng YinBayodé Roméo AdégbitèMeng ZhuBeata Butruk-RaszejaMeng-Chi YenBeata JabłońskaMeng-Han TsaiBeata KaletaMenglong XuBeata Mrozikiewicz-RakowskaMengxi ShenBeata OlejnickaMeraj Alam KhanBeata PajakMercè MasanaBeata Sarecka-HujarMercedes FernandezBeata Szulc-MusiołMercedes Mitjavila-CasanovasBeatrice MacchiMerica Glavina-DurdovBeatriz San-MiguelMerli SaareBebiana CondeMervin YoderBee Kang TanMervyn J. EadieBeenu Moza JalaliMetin UzBehnam VafadariMette Frahm OlsenBehrooz H. YousefiMi Kyung ParkBéla KovácsMichael A. FirerBelém Sampaio-MarquesMichael A. UrbinBelur R. LokeshMichael BukrinskyBen HamzaMichael BullardBenedetta E. FornasariMichael J. B. KutrykBenedikt EggerMichael J. CurtisBeniamin Oskar GrabarekMichael JaffeBenita WiatrakMichael KeusgenBenjamin E. Nilsson-PayantMichael KhalilBenjamín FloránMichael LandowskiBenjamin J. BurwitzMichael MaasBenjamin JurekMichael ModelBenno WeigmannMichael MorganBenny RyplidaMichael P. MarkeyBenoît MiottoMichael PedersenBerenika M. Szczȩśniak-SiȩgaMichael PinkawaBernadeta ChyrchelMichael RothBernat SoriaMichael S. WolinBernd StratmannMichael SchnoorBernd W. SiguschMichael SigalBernhard BiersackMichael SticherlingBernhard RauchMichael WeitzendorferBernhard RyffelMichael YoussefBernhard SpinglerMichail KlontzasBernhard WeberMichal A. RahatBert VandenberkMichał AntoszczakBertrand LiagreMichal J. DabrowskiBerwin Singh Swami VethaMichał J. SobkowiakBettina BlaumeiserMichal JaniakBetty Yuen-Kwan LawMichal JurášekBhakti BasuMichał KuszewskiBhakti PatelMichal LetekBhanu TewariMichal LinialBhasker RadaramMichal MaczewskiBhaswati BhattacharyaMichal OrdakBhavita WaliaMichal PyzikBhawik Kumar JainMichał RabijewskiBhoomika PatelMichał Stefan LachBi ZhaoMichal ZarobkiewiczBiagio BaroneMichal ZmijewskiBiagio DidonaMichel RoethlisbergerBianca NitzscheMichela FerrucciBibhuti B. DasMichela PozzobonBignardi MarioMichela RigoniBing YangMichele BernasconiBissera PilichevaMichele Cosimo SantoroBiswajita PradhanMichele D’AttilioBjörn TampeMichele Di CosolaBo WangMichele Di FoggiaBogdan DorofteiMichele FinottiBogdan IliescuMichele FioreBogdan Ionel TambaMichele LaiBogdan Ovidiu PopescuMichele MarchioniBogdan SevastreMichele SalemiBogdan Silviu UngureanuMichelle ThomasBogdan SoceaMichiko ShimodaBogusz Aksak-WąsMieszko WięckiewiczBoleslaw T. KarwowskiMigle BacevicieneBoMi RyuMiguel A. RubioBoncho KuMiguel HuesoBono LučićMiguel IdoateBoris KozlovMiguel MartinsBoris N. KhlebtsovMiguel OrtegaBorislav AngelovMiguel Ponce-VargasBorja Herrero De La ParteMiguel Segura GinardBor-Show TzangMihaela Adriana IlieBortolo MartiniMihaela HedeșiuBotond PenkeMihaela TurtoiBoya LiuMihai Cosmin CenariuBoyan GrigorovMihai MoldovanBozhidar StefanovMihai PopescuBrach PostonMi-Heon RyuBradicich MatteoMika O. RuonalaBrandon LewisMika SugaBrandon Lucke-WoldMikael S. LindströmBranislava MilenkovićMikhail G. AkimovBrenda LakyMikhail KislinBrenda LeeMiki KushimaBrian D. AdamsMikio HayashiBrian GabrielliMikó ÉvaBrian T. DavidMilan JakubekBrian WalittMilena GeorgievaBrigitte AltmannMilica MarkovicBrigitte HantuschMilica PešićBrijesh SinghMilos PjanicBrînduşa Alina PetreMiloslav MachacekBritt WildemannMineko TeraoBrittany MurphyMinerva Codruta BadescuBrunella GrigoloMing ChenBruno ChrcanovicMing Feng LiaoBruno FiondaMing Hsien TsaiBruno ImbimboMing YangBruno MeloniMingchong YangBruno PéaultMing-Feng WuBruno Vinicius Manzolli RodriguesMingfu ChenBryan FullerMinghui CaoBuddhadev PurohitMingjun TsaiBulent AhishaliMing-Kuei ShihBum-Rak ChoiMinglei GuoByoung Kuk JangMingli WangByron BaronMingming TongByung-Hoon JeongMingsong KangByungsuk KwonMing-Tsun TsaiC. Jane WelshMing-Yi ShenC. Michel ZwaanMin-Kyu KimCalado Cecília R. C.Minoo RassoulzadeganCălin CăinapMinoru GotohCambón AdrianaMin-Sik KimCamelia CoadaMinvydas RagulskisCamelia QuekMiodrag JanićCameron JohnstoneMiodrag LazarevićCamille CharbonnierMiodrag LovricCamino de Juan RomeroMir S. AdilCara WestmarkMira A. M. BehnamCarina BalcoşMiran RadaCarine MichielsMiran SebestjenCarl WeinerMircea TampaCarla PeregoMireia MallandrichCarla PetrellaMirela TiglisCarlo AprileMiren AltunaCarlo CervellatiMiriam HickeyCarlo PerisanoMiriam LongoCarlos AmeroMirjana JerkićCarlos ArámburoMirko KarakašićCarlos Cuenca-BarralesMirko ManchiaCarlos Eduardo Fonseca-AlvesMirko ManettiCarlos González-FernándezMirko VoelkersCarlos Martin LlorenteMiro JukićCarlos Martinez-CampaMiroslav BarancikCarlos Pérez PlasenciaMiroslava KačániováCarlos Pérez-MonterMiroslava RabajdovaCarlos Portugal-NunesMirosław AniołCarlos Rocha-de-LossadaMirosława Ferens-SieczkowskaCarlos TrenadoMiroslawa PuskulluogluCarlos Vicario-AbejónMiruna Silvia StanCarmela PaolilloMirza PojskicCarmela RinaldiMithalesh Kumar SinghCarmela SantangeloMititelu Tartau LilianaCarmelo BellarditaMitja KosnikCarmelo MilitelloMitsuru NakazawaCarmen BarticMitsushige SugimotoCarmen ClaroMiusi ShiCarmen De MiguelModesto De CandiaCarmen DomínguezMohamad NavabCarmen Méndez HernándezMohamed AbdelgiedCarmen Moret-TatayMohamed Al-FatimiCarmen PanaitescuMohamed Jawed AhsanCarmen RinaldiMohamed M. DarwishCarmen Sorina MartinMohamed MahdiCarmina Teresa FussMohamed ShehataCarmine ColucciniMohamed ZakyCarmine FinelliMohamed ZoughaibCarmine IzzoMohammad Abu YousufCarol LinMohammad Fahad UllahCarolina DonàMohammad Faheem KhanCarolina Francelin RovarottoMohammad MohammadianpanahCaroline HastingsMohammad QneibiCaroline Lei WeeMohammad Tahir SiddiquiCarrie StewartMohammad YosofvandCarsten ZeilingerMohammad Zakir HossainCaspar StephaniMohammed BarigouCassiano Felippe Gonçalves-De-AlbuquerqueMohammed DiykhCassie S. MitchellMohammed HawashCatalina MihaiMohammed Oday EzzatCatalina PicóMohandoss SonaimuthuCatarina Luís SilvaMohd Ahmar RaufCatarina S. H. JesusMohd ImranCatarina SeabraMohmed Isaqali KarobariCaterina BuffoneMoises DiagoCaterina Maria GambinoMona BatishCaterina PalleriaMona M. El-RefaeyCatherine YzydorczykMona M. FreidinCatriona KellyMònica AguileraCécile-Marie Aliouat-DenisMonica FrinchiCecilia BattistelliMonica MiozzoCecilia GiuliviMonica NavarroCecilia J. HillardMonika Astasov-FrauenhofferCecilia NalliMonika BarathovaCecilia VarjuMonika BłaszczyszynCelestino SarduMonika FranczukCélia Duarte CruzMonika GrymowiczCélia F. RodriguesMonika KniotekCélia M. AntunesMonika KoziełCéline J. GuigonMonika Łopuszańska-DawidCélio Geraldo Freire-de-LimaMonika Sakowicz- BurkiewiczCesar BorlonganMonika SzeligaCésar PicadoMontserrat MaríCesare C. BerraMoon Nyeo ParkCesare CamettiMoonkyoo KongCesare R. SirtoriMootaz SalmanCezary WojtyłaMorag LewisChafia Touil-BoukoffaMoran AmitChaido SirinianMorel Jean LucChakraborty SohiniMorena FasanoChalermchai KhemtongMorgan Durette SalmonChalermpong SaenjumMorihito TakitaChamara V. SenaratnaMorito KurataChanchal MandalMostafa WalyChandrakala Aluganti NarasimhuluMostefa Mohamed-SeghirChandrasekhar YadavalliMoustafa T. MabroukChang Won ChoiMucong LiChangqi LiuMuhammad A. AlsherbinyChangqing SuMuhammad A. JawadChao HeMuhammad ArsalanChaofan LiMuhammad KhurramChaoqun HuangMuhammad NawazChao-Yu GuoMuhammad RiazCharalampos SiristatidisMuhammad Shuja KhanCharat ThongprayoonMuhammad SohailCharis LiapiMuhammad UmerCharles BaileyMuhammad Yasir AsgharCharlotte LawsonMuhammad Zubair IqbalCharlotte von GallMuhammad Zubair IsrarChellappagounder ThangavelMukesh VermaChen-Chi WuMumtaz AnwarCheng HuMunawar NaylaCheng-Hsun ChiuMun’delanji VestergaardCheng-Shun LuMuneeswar NittalaCheng-Tien WuMunehisa ShinozakiChengwen SunMuneyoshi OkadaCheng-Yang HuangMurad MollaevChengyi TuMurali MunirajuCheorl-Ho KimMurray KorcChetna SoniMuruganandan ShanmugamCheuk Lun LiuMussie MsghinaChi Chun WongMuthaiah ShellaiahChia Shan WuMuthuramalingam PandiyanChiachi LiuMuyun XuChia-Chi WangMyriam CayreChia-Chun TsengMyriam RichaudChia-Hung ChouMyungsoo JooChia-Hung YenNabeel AhmedChiao-Yin SunNadezhda V. ShilovaChiara ArrigoniNadine Mestre-FrancésChiara BrulloNagaraja S. BalakathiresanChiara EandiNagendra Kumar KaushikChiara FoglieniNajeeb UllahChiara GiacomelliNalu Navarro-AlvarezChiara LiveraniNam-gu HerChiara Maria MazzantiNamsun PaikChiara MoltrasioNanlin WuChiara MottaNao HorioChiara ValoriNaoki IkegayaChia-Ti TsaiNaoki SatoChi-Chang YuNaoki TanakaChi-Chien LinNaoki UmemuraChichun HoNaoro TanakaChien-Chin ChenNaoyuki KataokaChien-Feng LiNarasimman GurusamyChien-Ming ChaoNarendar DudhipalaChih-Cheng LaiNarendra Nath JhaChi-Hung LinNarendranath EpperlaChih-Yang WangNaresh KumarChih-Yao HouNaro OhashiChih-Yen ChienNarongchai AutsavaprompornChin Wei LaiNatalia A. IgnatenkoChingfeng WengNatalia A. MuralevaChinghsiung LinNatalia AbramenkoChing-Hui SiaNatalia BelosludtsevaChing-Jin ChangNatália Cruz-MartinsChin-Kun WangNatalia Díaz-ValdiviaChinmoy PatraNatalia FilippovaChin-Mu HsuNatalia GrubaChinnadurai ManiNatalia IssaevaChip HahnNatalia LisiakChisato OheNatália MartinsChi-Ting HorngNatalia NaryzhnayaChloe De RooNatalia RiveraCho Yeow KohNatalia SavelyevaCho-Hao LeeNatalia ShnayderChongxiao ChenNatalia SzejkoChris BusbyNatalia V. GulyaevaChris H. L. LimNatalia Viktorovna NizyaevaChris Van Der PoelNatalie H. MatthewsChrishan J. A. RamachandraNatalie M. ZahrChrista BüchlerNatalija FilipovićChristel NeutNataliya KolosovaChristian BarbatoNataliya Valentinovna YaglovaChristian BehmNatalja EigelieneChristian BleilevensNatassa PippaChristian F. FreyschlagNatella EnukashvilyChristian LegrosNathalia PizatoChristian M. H. R. De GeyterNathalie BardinChristian MorsczeckNathan GallantChristian TanislavNathan K. EvansonChristian VergaraNathan L. AbsalomChristian WunderNathan LanningChristie Ying Kei LungNattawat KlomjitChristina Anna StratopoulouNayden G. NaydenovChristina CoughlanNebojsa MalesevicChristina E. KostaraNeda SladeChristina PamporakiNeelesh Kumar MehraChristoph BuchtaNeha NandaChristoph DanielNehaben A. GujaratiChristoph JochumNeil YoungsonChristoph ReinhardtNejc UmekChristophe DeroanneNela MalatestiChristopher AikenNela PivacChristopher D. PoradaNeli Kachamakova-TrojanowskaChristopher EllisNelofer SyedChristopher GrothNenad VukojevićChristopher HillNerea Cuesta-GomezChristopher J. SmithNerea Iturrioz-RodríguezChristopher KobierzyckiNeven PapicChristopher V. HughesNeven ZarkovicChristopher W. VaughanNguyen Minh DucChristopher W. WassonNiall MurphyChristos K. KontosNicha PuangmalaiChristos PapaneophytouNicholas G. JamesChrysa VoidarouNicholas G. KounisChuanbin MaoNicholas J. ThomasChuanming XuNicholas P. IllsleyChulhwan ParkNicholas P. RowellChun GuoNicholas PeakeChun Hung ChuNick KouttabChun Nam LokNicola FerriChun-Chia ChengNicola LongoChung-Jung LiuNicola MadugnoChung-Min WuNicola MondanelliChung-Wei KungNicola OreficeChunhui LiNicola OrigliaChunliu ZhuoNicola PederneschiChun-Ru WangNicola ScottiChunying LiNicola SilvestrisChutima KunacheewaNicola ValenteCinzia RotondoNicola ZampieriCiprian TomuleasaNicolae BacalbașaCiro De LucaNicolas BeryCiro Gargiulo IsaccoNicole L. BatenburgCiro ImbimboNicole SommerCiro Pasquale RomanoNicoleta Nicole RaduClaire DebackNicoletta PedemonteClaire DemiotNicolina CapoluongoClaire E. Kendal-WrightNicolò MussoClaire StockerNicolòmaria BuffiClara O. SailerNidhi Jalan-SakrikarClare HoskinsNiels Frimodt-MøllerClare SchillingNiels Nørskov-LauritsenClarissa Fantin CavarsanNiels QvistClaude Caron De FromentelNik TsotakosClaude Kwe YindaNikesh GuptaClaudia BerneckerNikhil Suresh BhandarkarClaudia DalmastriNikhilesh JoardarClaudia DesiderioNiko SillanpääClaudia DurantiNikola GolenhofenClaudia Ferreira Da Rosa SobreiraNikolaos A. ChrysanthakopoulosClaudia KitzmüllerNikolaos AntonakopoulosClaudia KusmicNikolaos E. PatsoukisClaudia MancaNikolaos MachairasClaudia Maria HattingerNikolaos PistamaltzianClaudia MartiniNikolas DovrolisClaudia MatteucciNikolay A. PyataevClaudia MehedintuNikolay V. GoncharovClaudia NastasiNikoleta G. PrintzaClaudia Ollauri-IbáñezNikos ViazisClaudia RicciNilakshi BaruaClaudia Staab-WeijnitzNilesh SharmaClaudinei dos SantosNilo RivaClaudio BrassoNina FilipczakClaudio de LuciaNina PastorClaudio GambardellaNina RembiałkowskaClaudio HumeresNing QuClaudio LuchiniNino RussoClaudio SorioNiraj NepalClaudio TabolacciNirakar SahooClaudio TostoNirmal K. RoyClemens Von SchackyNitin AmdareClemens ZwergelNitin KhandelwalClement MesedaNobuari TakakuraClementina ManeraNobuko HosokawaCleydson Breno Rodrigues dos SantosNobumichi KobayashiClio P. MavraganiNobuo OkumuraClive KellyNobuyuki KimuraColleen CronigerNoe B. MercadoCongbao KangNoemí Cárdenas-RodríguezCongcong ZhouNoemi DirzuConghui LiuNoor ZamanConnor BamfordNoortje De HaanConstantino Carlos Reyes-AldasoroNora MöhnConsuelo Cháfer-PericásNorbert GrafConxita MestresNorbert KissCoralia BleotuNorbert StefanCorin MillerNorbert SzentandrássyCornel BaltăNoriaki IwahashiCorneliu MunteanuNoriaki YoshidaCorrado AngeliniNorihiko NaritaCorrado CampochiaroNoriko HiraishiCorrado RomanoNoriko TakegaharaCorrado TagliatiNuno FrancoCosimo BruniNuno ValeCosmin EneNunzia IaccarinoCosmin Mihai VesaNunzia MatroneCostantino BalestraNunzio VicarioCraig BlackstoneNuria MorralCrescenzio SciosciaNurit HaspelCristian BersezioNury Pérez-HernándezCristian I. SurcelOana CadarCristian MunteanuOctavian AndronicCristian SmerdouOctavian EnciuCristiana GonçalvesOctavian Tudorel OlaruCristina AchimOhad MazorCristina DaiaOke GerkeCristina FasolatoOladapo BakareCristina FerrerasOlaf GriskCristina GluhovschiOlan Jackson-WeaverCristina Lavareda BaixinhoOlatz ZenarruzabeitiaCristina M. FaillaOleg A. RakitinCristina MambetOleg N. TimofeevCristina Martin-SabrosoOleg ShuvalovCristina MennittiOleksii TyshchenkoCristina OrtegaOlesya V. StepanenkoCristina PiñaOlga AnatskayaCristina RodríguezOlga Catalina RodriguézCristina Sánchez-De-DiegoOlga Czerwińska-LedwigCristina SatrianoOlga FedorovaCristina SegoviaOlga GolubnitschajaCristina SoléOlga GordeevaCristoforo PomaraOlga KopachČrtomir PodlipnikOlga KorczeniewskaCsaba HarasztosiOlga LebedevaCynthia BlantonOlga M. BazanovaC-Yoon KimOlga M. Koper-LenkiewiczCyrus MotamedOlga MatveevaD. S. PrabakaranOlga S. TarasovaDagmar PavlůOlga Yurevna SelyutinaDagmara Wróbel-BiedrawaOlga ZaborinaDahang YuOlivera Casar-BorotaDaiki TaniyamaOlivera Mitrović AjtićDalal Hammoudi HalatOlivia DorneanuDa-Liang OuOlivier BousigesDalila ScaturroOlivier RousselDamien ArnoultOlle RingdénDan PredescuOlof ErikssonDan QiOmar CauliDana Gabriela FestilaOmar El BounkariDana StoianOmar El HibaDana Teodora Anton-PaduraruOmar KujanDaniel C. ChristophOmero Bendicto Poli-NetoDaniel ElbirtOmid AkhavanDaniel EllederOndrej KodetDaniel GeorgeOndřej SlanařDaniel Gonçalves-CarneiroOndrej VasicekDaniel J. RawleOphir NaveDaniel José BarbosaOriana Lo ReDaniel KronenbergOriana TrubianiDaniel L. HerrOriella GennariDaniel López MaloOrjena ŽajaDaniel MartinOrsola Di MartinoDaniel NeureiterOsamah Al RugaieDaniel Ortuño-SahagúnOsamu IshidaDaniel PinkOsamu KumanoDaniel PotaczekOscar CasisDaniel RoethOskar SachenkovDaniel SteinOskars KalejsDaniel TurudićOtávio EspíndolaDaniel VaimanOttilie LoeffelholzDaniel Xin ZhangOttmar HerchenröderDaniela Amidžić KlarićOvidiu Alexandru MederleDaniela C. PaseroOvidiu MituDaniela CaccamoOvidiu PopDaniela CalinaOwen LeiserDaniela Carmen AbabeiOxana M. DrapkinaDaniela IannazzoOzgul GokDaniela Maria TanaseOzgur BasalDaniela MarzioniOzlem Ipek Kalaoglu-AltanDaniela MiricescuOzren PolašekDaniela Opris-BelinskiPadulo JohnnyDaniela PintoPalaniraja ThandapaniDaniela TerraccianoPalash SanphuiDaniele BaniPallavi ShrivastavaDaniele CavaleriPamela Di GiovanniDaniele CorboPanagiotis AthanassiouDaniele GarcovichPanagiotis ChristopoulosDaniele MasaronePanagiotis GeorgianosDaniele SolaPanagiotis GkriliasDaniele Ugo TariPanagiotis MallisDaniele VergaraPanagiotis TheodorakisDanieli AndradePanagiotis TheofilisDanielle GreenbergPandurangan RamarajDanijela BarićPaniz JasbiDanijela Vrdoljak MozeticPankaj AhluwaliaDanila de VitoPankaj GaurDanilo Martins Dos SantosPanos LoukopoulosDanilo VonaPantelis KourosDanko MiloševićPantelis ZempekakisDanny MisiakPaola De LucaDanny Van Der HelmPaola Di PietroDaoying GengPaola FerrariDapeng ChenPaola ImbriciDaria AugustyniakPaola ManiniDaria DanilenkoPaola PiomboniDarin ZertiPaola PontrelliDario BrunettiPaola QuaresimaDario Di NardoPaola RusminiDario SiniscalcoPaola SavoiaDario-Lucas HelbingPaola TuranoDariusz KałkaPaola UngaroDariusz Maciej PisklakPaola ViganòDariusz NowakPaôline LaurentDarrell CockburnPaolo BennaDarya NovopashinaPaolo CameliDave GilmorePaolo CampioniDavid A. HartPaolo CappareDavid Alexander Christian MessererPaolo FagoneDavid Arranz-SolísPaolo GasparellaDavid B. AlexanderPaolo ImmovilliDavid BalayssacPaolo MarracciniDavid Bar-OrPaolo MeneguzzoDavid CantusPaolo MonardoDavid ChambersPaolo SpinnatoDavid Changli WeiPaolo Tranquilli LealiDavid CharlesPaolo TrucilloDavid D. BushartPaolo TucciDavid DolivoParamita ChakrabortyDavid DoraParamjit S. TappiaDavid EscorsParaskev T. NedialkovDavid F. DelotterieParaskevi KarousiDavid G. CoffeyParisa GazeraniDavid G. GreenhalghParmjit S. JatDavid GerberParvesh WadhwaniDavid GrahamParvez AlamDavid Harry McDougalPaschalis TheotokisDavid HoogewijsPasquale EspositoDavid L. CarawayPasquale MastrorobertoDavid LeavesleyPasquale PisapiaDavid MorrisPasquale ScarciaDavid Orlando Alves FerreiraPasqualino SirignanoDavid PagliaPatrícia AlvesDavid PerpetuiniPatricia J. LardoneDavid RoyPatricia K. CoyleDavid Scott McVeyPatricia M. LeglerDavid VětvičkaPatrick AugusteDavide BarbagalloPatrick ButayeDavide BertelliPatrick DansetteDavide BorroniPatrick G. ArndtDavide LauroPatrick LauDavide MoiPatrick NasarreDavide MugettiPatrick WeydtDavide PorrelliPatrizia AgrettiDavide RaineriPatrizia ManciniDavood KharaghaniPatrizia PorcuDawei JiangPatrizia ZaramellaDawid MadejPatrycja KleczkowskaDawid PrzystupskiPatrycja KupnickaDawid SigorskiPaul BălănescuDawn QuellePaul E. LoveDe Gaulle I. ChigbuPaul F. WilliamsDebajit DeyPaul GardDebananda GogoiPaul GissenDebasish Kumar DeyPaul GooleyDebolina BiswasPaul PotterDebora ColangeloPaula Alexandra PostuDeborah CroteauPaula HoffDeclan McKernanPaula HoffmanDeepa AcharyaPaula J. JauregiDeepak Kumar KhajuriaPaula KeschenauDeepak VangalaPaula Mello De LucaDeependra Kumar SinghPaula TavaresDeepika AwasthiPaul-Mihai BoarescuDeepika GargPaulo A. GameiroDeguan LvPaulo Jorge PalmaDejan JakimovskiPaulo Magno Martins DouradoDelia Ioana HorhatPavan Kumar DhanyamrajuDemetrios A. ArvanitisPavel A. ShilyaginDemitrios VyniosPavel GotovtsevDenis BourgeoisPavel MarešDenis BuxtonPavel SolopovDenis N. SilachevPavel ZhabyeyevDenis S. KudryavtsevPaweł GaćDenise Fernandez-TwinnPawel KleczynskiDenise HemmingsPaweł KordowitzkiDenise ToscaniPaweł KowalczykDennis EurichPaweł KwiatkowskiDennis NiebelPawel OlczykDennis V. CokkinosPaweł ŚwitDeo SinghPaweł WańkowiczDesiree WandersPaweł WeichbrothDésirée WünschPayal GangulyDespina KalogianniPayaningal SomanathDessie Salilew-WondimPedro BullonDesu ChenPedro De-la-TorreDetlef ObalPedro Ferreira-SantosDevakar EpariPedro J. CabralesDezso KelemenPei Yuin KengDhananjay YadavPeichao ChenDhanush HaspulaPeifang SunDi WuPei-Yi ChuDi ZhangPeng LiDiana Andreia Tavares PintoPeng LiaoDiana Camelia NuţǎPeng ShaoDiana CenariuPeng WeiDiana Cláudia PintoPenny A. RuddDiana Cunha-ReisPernille HolmagerDiana MartinsPershina OlgaDiana MechtcheriakovaPetar OzretićDianjun CaoPetca Razvan-CosminDidar AsikPeter CelecDidier MerlinPeter D. WestenskowDidier PicardPeter JonesDiego F. CalvisiPeter KorstenDiego Franco JaimePeter MúdrýDiego La MendolaPeter PensonDiego RaimondoPeter Physick-SheardDillon MintoffPeter VeldhuizenDimiter KunnevPeter VerwilstDimitrios FilippiadisPēteris TretjakovsDimitrios GougoulisPeter-Paul WillemseDimitrios KapsokalyvasPetr O. IlyinskiiDimitrios KaragiannakisPetra KoraćDimitrios KotsirisPetras PrakasDimitrios TzachanisPetronella KettunenDimitrios VlachodimitropoulosPetros P. SfikakisDimitrios VrachatisPhil AyersDimitris TatsisPhil WhitfieldDimitris ZacharoulisPhilip D. ChilibeckDina Costa SimesPhilip H. G. ItuarteDipankar AshPhilip J. YoungDirk GrimmPhilip WertzDirk H. OrtgiesPhilippe GermainDirk HecklPhilippe PourquierDirk MontagPhilippe R. KoninckxDirk Van WestPhilippe RocheDivya MurthyPier Adelchi RuffiniDivya SinhaPier Lorenzo PuriDjida GhoubayPier Luigi MeroniDjuro JosicPier Paolo PiccalugaDmitrii A. TanianskiiPiera Di MartinoDmitry KarpovPierangela TottaDmitry KhochenkovPierfrancesco TassoneDnyaneshwar RasalePierfranco ConteDolores Ortiz-MasiáPiero PaladiniDomenico AlbanoPierre DeharoDomenico De BerardisPierre JoukDomenico Edoardo Pellegrini-GiampietroPierre Schembri WismayerDomenico LioPierre-Yves Collart-DutilleulDomenico MaioranoPietro FerraraDomenico MontesanoPietro MesircaDomenico RussoPietro ScicchitanoDomenico TricaricoPinelopi SamaraDominik DłuskiPing SongDominik ŁagowskiPing WangDominika GłąbskaPing-Hsing TsaiDominika SalamonPiotr B. HeczkoDominika StygarPiotr ChmielarzDominique HooglandPiotr DobrowolskiDominique ManikowskiPiotr DuchnowskiDominique ModrowskiPiotr DzięgielDonatella PontiPiotr MichelDonato ColangeloPiotr MuchaDong HanPiotr PoznanskiDongchuan GuoPiotr ZapalaDongdong LuPius N. NdeDongdong WangPiyush BaindaraDong-Jin LimPlamen KatsarovDong-Woo ChoPolly LeungDong-Wook HanPorin PerićDongyue XinPoungrat PakdeechoteDonna WallPovilas IgnataviciusDoreen LauPrabaha SikderDorinda Marques-da-SilvaPrabakaran NagarajanDorota NieoczymPrabhakar BusaDorota SzczȩsnaPrabhu RamamoorthyDorota Tichaczek-GoskaPrabhugouda SiriyappagouderDorota ŻelaszczykPrabhuraj D. VenkatramanDorothée Kasteleijn-Nolst TrenitéPradeep Kumar BollaDouglas Maya-MilesPradeep SinghDragana KomnenovPradoldej SompolDragana Mitić-ĆulafićPrakash GangadaranDragos SerbanPrakash KshirsagarDriss CherqaouiPranabananda DuttaDrozdstoi StoyanovPrasannavenkatesh DuraiDubravka Švob ŠtracPrasanta DashDubravko HabekPrasanth PuthanveetilDuo ZhangPrasert AuewarakulDurai SellegounderPrashant KumarDylan LeiPrashanth N. SuravajhalaDylan RichardsPrashanth NageshDzung H. DinhPrathap SomuEason HildrethPratiti BhadraEberhard UhlPrawej AnsariEbtesam Al-SuhaimiPrem YadavEddie A. JamesPritesh P. JainEdgar A. JaimesPriyanka ShawEdgar Tavares-da-SilvaProttoy HasanEdmunds ReineksPrzemyslaw ChmielewskiEdoardo BraunerPrzemysław KorohodaEdoardo MelilliPrzemysław SitarekEdoardo MilanettiPugazhendhi SrinivasanEdoardo PasquiPuja KumariEduard KorkotianPuran S. BoraEduardo ArtalPushpa Raj JoshiEduardo Domingos Correia GomesPuxiang LaiEduardo Fuentes QuinterosPyotr A. SlominskyEduardo Gutiérrez-AbejónQi JinEduardo Pandolfi PassosQi ShaoEduardo Rivadeneyra-DomínguezQian DuEdvard EhlerQiang HuangEdvardas DanilaQiang LiuEdward BormashenkoQianqian LiEdward JudeQian-Xiong ZhouEdward KoifmanQiao ShiEdward ZimbudziQibin GengEdyta Ewa SutkowskaQingming FangEdyta MakuchQingyu ZhouEfraín Santiago-RodríguezQinyu HaoEfstathia K. KapsogeorgouQiong ZhangEfstratios-Stylianos PyrgelisRabiul IslamEgor Y. PlotnikovRachel EigesEhsanul Hoque ApuRachel OldershawEiichi SatoRachele IsticatoEiichi WatanabeRachy AbrahamEiichiro NagataRadmila JankovićEiji KiyoharaRadoslav MatejEiji KubotaRadosław ChaberEijiro JimiRadosław KujawskiEirini ChristodoulouRadosław MaksymEirini VasarmidiRadoslaw Marek KiedrowiczEisa Tahmasbpour MarzouniRadosław WolniakEishin MoritaRadu C. RacovitaEitan FibachRadu ChiceaEitaro KodaniRafael EscateEjay NsugbeRafał BielasEjaz A. AnsariRafal DankowskiEkaterina RudnitskayaRaffaele D’AmelioEkaterina V. SeminaRaffaele De CaroEkaterina YurchenkoRaffaele PalmirottaEleftherios KavroulakisRaffaele SerraElena A. JonesRaffaella BrugnoniElena AndreucciRaffaella CascellaElena BazhanovaRaghava PotulaElena BernadRaghunath SinghElena BoggioRahul KumarElena CodriciRahul MannanElena CsernokRahul SrinivasanElena De FalcoRais Ahmad AnsariElena De FeliceRaj KumarElena DinteRaj Kumar MongreElena FicoRaja GanesanElena GubarevaRaja KalluruElena I. RyabchikovaRajakrishnan VeluthakalElena Iuliana BîruRajamma MathewElena M. KondaurovaRajeev K. SinglaElena Martens-UzunovaRajesh K. TripathyElena MikhalchikRajesh KasamElena Obrador PlaRajesh ParsanathanElena PopugaevaRajesh SharmaElena ShramovaRajesh SinghElena TarcaRajeshwary GhoshElena TchetinaRaji P. GrewalElena UspenskayaRajia BahriElena ZavyalovaRaka JainElena ZenaroRalph BuchertEleni AnastasiadouRalph FingerhutEleonora AricòRaluca Simona CostacheEleonora MargheritisRam SagarEleonora RussoRamana VakaEleonóra SpekkerRamandeep S. TakhterEleonore FröhlichRamar ThangamEleuterio A. Sanchez RomeroRamasatyaveni GeesalaElham PishavarRamesh ChingleElia BariRamesh KandimallaEliana GianolioRamit Maoz-SegalEliana ScemeRamona D’AmicoElias A. KouroumalisRamona PalomboEliecer CotoRania AbutarboushElisa AraldiRanjana MitraElisa BelluzziRaphael CiumanElisa CabiscolRaquel Benedé UbietoElisa CairraoRaquel BoiaElisa Lopez-DoladoRasika SamarasingheElisa OltraRasoul GhasemiElisabete Cristina Nunes FernandesRaúl Díaz-MolinaElisabete M. S. CastanheiraRavi Chakra TuragaElisabeth PinartRavi P. SahuElisabeth ZechendorfRavi Philip RajkumarElisabetta CatalaniRavinder KumarElisabetta FerraraRavindra DeshpandeElisabetta FrattaRavindran Caspa GokulanElisabetta GiorginiRavindranadh KoutavarapuElisabetta ProfumoRaviraj VankayalaElise Gomez-SanchezRaymond Scott TurnerElizabeth BowdridgeRazique AnwerElizabeth M. MartinRăzvan Adrian IonescuElizabeth ThomasRazvan SocolovElizabeth VafiadakiRazvan Stefan BoiangiuElizabeth YehRăzvan-Ionuț PopescuElla GrilzRebeca BustoElvira IngleseRebecca AppletonElvira RozhinaRebeka ZsoldosElvire Pons-TostivintReem Smoum-JaouniElzbieta JandaReem T. AtawiaElżbieta Wałajtys-RodeRegina M. DayEma OzakiRegina WierzejskaEman A. ToraihReginaldo G. BastosEman MehannaRégulo López-CallejasEmanuela ChiarellaRehan KhanEmanuela LocatiReinhard Schweitzer-StennerEmanuele BernardinelliReinhard ZelenyEmanuele CarnielReinis VilskerstsEmese MezõsiRéjean CoutureEmiel F. M. WoutersRemco J. MolenaarEmiko SenbaRemedios Lopez-LiriaEmil BulatovRémi LonguespéeEmilia VassilopoulouRemy PoupotEmiliano DallaRenan Pedra De SouzaEmilio ParisiniRenata BalnytėEmilly Schlee VillodreRenata MikolajczakEmily S. J. EdwardsRenata Novak KujundžićEmina Emilia TorlakovicRenato Alberto SinicoEmma C. CacciolaRené HägerlingEmma MontellaRene VidalEmmanuel N. KontomanolisRené WenzlEmmanuel Navarro-FloresRené WubbelsEmmanuel RaffouxRenee J. ChosedEmoke PallReneta GevrenovaEmöke ŠteňováReza PiriEndre ZimaRicardo DiasEndrit ShahiniRicardo VardascaEnqi LiuRiccardo CristofaniEnquan XuRiccardo IentileEnrico AmmiratiRiccardo MonterubbianesiEnrico BraccoRiccardo TelliniEnrico CarminaRicha SachanEnrico GarattiniRichard A. AmosEnrico RadaelliRichard C. DowellEnrico VelardiRichard G. FesslerEnrique SotoRichard IvellEnrique VerdúRicia Katherine HydeEny Maria Goloni-BertolloRiedemann ThereseEpaminondas DoxakisRiëm El TahryEphraim WinocurRiham FliefelEran TauberRiikka MartikainenErdem GökerRika MaruyamaErden AtillaRikke KruseEric A. EppingRiku KawasakiEric De GrootRinesh GodfreyEric HajduchRinkoo Devi GuptaEric MasRipon SarkarEric Minwei LiuRishabh DevEric Scott SillsRishil KathawalaErica BazzanRisto A. KauppinenErica VetranoRita BellaErica ZamberlettiRita De Cássia Pontello RampazzoErich MöstlRita KissErik BehringerRita PavasiniErik Martínez-HackertRita SattlerErik SauleauRitesh RamdhaniErika A. EksiogluRitesh SrivastavaErika Bevilaqua RangelRizwan WahabErika CioneRob T. StrikerErika L. NurmiRobert C. LiuErika M. PalmieriRobert DaileyÉrika Said Abu EgalRobert DraguErin Elizabeth SchirtzingerRobert DrillienErkko O. YlösmäkiRobert EckenstalerErmelindo C. LealRobert GniadeckiErmolenko E. I. ErmolenkoRobert GouldErnest AdeghateRobert J. PalmerErnesto D’AlojaRobert KammererErnesto GargiuloRobert KissErwin BrosensRobert L. MeiselEsmail DoustkhahRobert MaittaEsrat Jahan RupaRobert NolandEssa SaiedRobert RusinaEssam H. HousseinRobert S. BenjaminEstanislao NavarroRobert SochaEstefanía Burgos-MorónRobert W. SigginsEstefania Lozano-VelascoRobert WessellsEstefania NuñezRobert Y. L. WangEstefanía TarazónRobert Yung Liang WangEstela SangüesaRoberta AngioniEster GuausRoberta BortolozziEsther MorenoRoberta FuscoEswar ShankarRoberta ImperatoreEtienne HerzogRoberta MacrìEtienne MoussayRoberta MoschiniEtsuro BandoRoberta PalmourEugene PostnikovRoberta PeruzzoEugenia BezirtzoglouRoberta PintusEugênio Gonçalves De AraújoRoberta ZupoEui-Bae JeungRoberto BenelliEun Jeong ParkRoberto BianchiEun Kyung LeeRoberto ChimenzEunate Gallardo-VaraRoberto CirocchiEunhee ChungRoberto CodellaEunju KangRoberto ColluEunyoung HaRoberto CorizzoEusebio ChiefariRoberto GramignoliEva AusóRoberto Li VotiEva BagyinszkyRoberto Martínez BeamonteEva CoreyRoberto MontaltiEva de Lago FemiaRoberto PaganelliEva KatonaRoberto PasseraEva M. CarroRoberto SaiaEva María Martínez-JiménezRoberto ScicaliEva Mischak WeissingerRoberto ZefferinoEva RamosRobin KumarEva ŠatovićRobin L. CooperEva TsaousidouRocchetti VincenzoEva TvrdáRocío Guzmán RuizEvalyn E. A. P. MulderRocio Nuñez-CalongeEvangelia EmmanouilidouRocío Ruiz LazaEvangelia VouvoudiRoddy HiramEvangelos KoustasRodica OlarEvanthia BernitsasRodney G. BowdenEvelina Maria GosavRodolfo IulianoEvelyne SchvoererRodrigo CunhaEvert F. S. Van VelsenRodrigo FerreiraEvgenia SitnikovaRodrigo Herrera-MolinaEvgeny BelyavskiyRoel Van OvermeireEvgeny E. BezsonovRoger BiringerEwa FiedorowiczRoger E. ThomasEwa Gibuła-TarłowskaRoger GrandEwa LaskowskaRohit KumarEwa TomaszewskaRohollah NasiriEwelina DziurkowskaRok FrlanEwelina KrólRok GaspersicEwelina PerdasRolf HeumannEytan R. BarneaRolf TeschkeFabian B. FahlbuschRomana VulturarFabian ItelRomeo T. CristinaFabiana GeraciRon KedemFabiana LucàRona GrahamFabiano CapparucciRonald A. LubetFabien ChevalierRonald B. BrownFabien PlissonRonan MurphyFabio Camacho-AlonsoRong-Fu ChenFabio CavaliereRonit ElhasidFabio GentiliniRonit SionovFabio GuoloRosa M. MartiFábio Juner LanferdiniRosa María AyalaFabio MonzaniRosa María Giráldez-PérezFabio PaceRosa SuadesFabio SantacaterinaRosalba GornatiFabrizia BonacinaRosalba ParentiFabrizio GentileRosalba SiracusaFabrizio MontecuccoRosalia BattagliaFabrizio SannaRosalia CrupiFahad KhanRosalinda SorrentinoFahd AlhamdanRosanna Di PaolaFaiyaz ShakeelRosaria MeccarielloFang HouRosario Francesco DonatoFang ZhengRosella CataldoFani LadomenouRosemary DziakFanny LegrandRosemary WalzemFa-Po ChungRoser López-AlemanyFarah AmmousRoser SolansFarid KhalloukiRossella CacciolaFarid RahimiRossella TuplerFarooq SyedRoubini ZakopoulouFarzad SalehpourRoxana Liana LucaciuFarzaneh SabbaghRoxana-Maria AmărandiFatemeh LavaeeRoyce CliffordFatih DemirRuben C. PetreacaFatima O. MartinsRuben MedinaFatimah KhalafRubén San-SegundoFaustine WilliamsRubina ShaikhFausto MaffiniRuchire WijesingheFederica BoschettiRudolf OehlerFederica CherchiRui VitorinoFederica De CastroRuitu LyuFederica FogacciRukmani PandeyFederica GuffantiRumiana TzonevaFederica ManninoRuosen XieFederica Morani Ruoxing WangFederica RomanoRupendra ShresthaFederico BardazziRupesh Kumar ChikaraFederico RavaioliRuslan MariychukFederico VancheriRussell D’SouzaFei PanRute SantosFeipei LaiRuth HalabanFelice CrocettoRuwei LinFelice LorussoRuža FrkanecFelice PetragliaRwik SenFelicia Gabriela GligorRyan McGrathFelipe Garrido Martinez de SalazarRyoichi AsakaFelipe Javier Chaves-MartinezRyoma MichishitaFelipe Martinez-PastorRyong Nam KimFelix BroeckerRyszard GrendaFelix GoniRytis MaskeliūnasFelix Javier Jiménez JiménezRyuichi SugiyamaFelix Martin WernerRyuji HayashiFelix SternbergRyutaro AsanoFelix YemanyiS. M. Shatil ShahriarFeng DongS. Priya NarayananFeng Liu-SmithSaad AlghamdiFeng TaoSaad HussainFeng XuSabata MartinoFengchao LangSabata PiernoFerdinando AgrestaSabine Kuchler-BoppFerdinando FranzoniSabine MazerbourgFerdinando PaternostroSabrina BossioFerenc DomokiSabrina LisiFernando CardonaSachchida Nand RaiFernando FerreiraSachiko MinamiguchiFernando Lopitz-OtsoaSachin VermaFernando MendesSachio FushidaFernando Padovan-NetoSachio TakenoFilipa PiresSadeq VallianFilippe Falcao-TebasSaeid Najafi FardFilippo BasagniSagrario Martin-AragonFilippo MarianoSahar KeshvariFilippo PiacentinoSahil InamdarFilippo TorrisiSai BiFilippos KoinisSaifei LeiFiorenzo MoscatelliSai-fu FungFiras KobeissySaiju JacobFiroz AkhterSaima ZameerFlavia Fondevila PeñaSajad KhazalFlávio FigueiraSajjad ShiraziFlavio RizzolioSalvador IborraFlemming AndersenSalvador PérezFlor H. PujolSalvatore Andrea MastroliaFlora N. BalievaSalvatore CocuzzaFlorence VorspanSalvatore De RosaFlorenci V. GonzálezSalvatore FeoFlorencia RosettiSalvatore SacconeFlorent BarbaultSalvatore SiracusanoFlorian BöhrnsenSalvatore Vincent PizzoFlorian GesslerSalwa El TawilFlorian KraftSamantha BartonFlorian KühnSamantha S. SoldanFlorina M. BojinSamar MansourFortunata CarboneSameera AbeyrathnaFotios DimitriadisSameh ElhadyFotis E. PsomopoulosSameh GehaFouquet GuillemetteSamet ÖzdemirFranca FagioliSami AkbulutFrancesca BianchiniSami H. TuffahaFrancesca BruzzeseSamia ElseginyFrancesca Chiara ViazziSamit ChakrabartyFrancesca De FeliceSamrita DograFrancesca DiomedeSamuel E. AdunyahFrancesca GuarinoSamuel HuberFrancesca OppedisanoSamuel Ken-En GanFrancesca PacificiSamuel Martins SilvestreFrancesca ReggianiSamuel ShiFrancesca RicciSamuele TarditoFrancesca Romana FuscoSandeep K. MishraFrancesco AsciSandeep Kumar Reddy AdenaFrancesco BertoliniSandeep MalampatiFrancesco BrunoSandesh PanthiFrancesco CavaniSandi KweeFrancesco CinettoSandip N. ChavanFrancesco ClapsSandor HosztafiFrancesco CoreaSandra BarbalhoFrancesco D’AlòSandra DonniniFrancesco De ChiaraSandra GhelardoniFrancesco Di GennaroSandra LeiszFrancesco FaitaSandra P. NunesFrancesco FerriniSandra PintoFrancesco GaziaSandra RebeloFrancesco InchingoloSandra ReznikFrancesco M. PacificoSandra ZampieriFrancesco ManfrediSandrine HormanFrancesco MannelliSandrine LauranceFrancesco OrziSangbum ParkFrancesco PepeSangjune KimFrancesco PerticoneSanja Popović-GrleFrancesco PivaSanjay DeyFrancesco PrattichizzoSanjay GuptaFrancesco RicchettiSankar JagadeeshanFrancesco ScavelloSanti MandalFrancesco SpinelliSanti NonellFrancesco StiloSanti SeguíFrancesco TovoliSantiago Navarro LedesmaFrancesco VasuriSantina Di BellaFrancis William LuscinskasSantos CastañedaFrancisco Alejandro Lagunas-RangelSantosh PothulaFrancisco ArnalichSaptarshi RoyFrancisco Carlos Pérez-MirallesSara BeneditoFrancisco Cortés-BenítezSara BozziniFrancisco G. Véliz-DerasSara C. ZapicoFrancisco Guillen-GrimaSara CorreiaFrancisco Lázaro-DiéguezSara DamianoFrancisco LeonSara De MartinFrancisco MartinsSara I. Al-GhadbanFrancisco Navarro-TriviñoSara PietracupaFrancisco Rodríguez-EsparragónSara RavaioliFrancisco-Javier González-BarcalaSara Remuzgo-MartínezFranciszek GłówkaSara SalatinFranck ChiappiniSara TyeballyFranco BassettoSarah AllegraFranco CervellatiSarah DuinFrançois BerthodSarah LaMereFrançois CornelisSaral DesaiFrancois EyskensSarit PalFrançois IchasSarmistha TalukdarFrançoise DegoulSaroj AmarFrane PaicSaša FrankFrank ComhaireSasha E. StantonFrank FischerSashi DebnathFrank RichterSathish Kumar NatarajanFrantisek LiskaSathish ThirunavukkarasuFranz ZehentmayrSatish MehtaFranziska SchmidtSatoru KobayashiFrédéric CoutantSatoru MunakataFrédéric EbsteinSatoshi TanakaFrédéric GoormaghtighSaturnino Marco LupiFrederic TortSaurabh MishraFrederick J. FullerSaurabh PandeyFrederik DenormeSaveg YadavFrederik PetersSaverio CandidoFredrick J. RosarioSaverio MuscoliFriedrich AltmannSavio Domenico PandolfoFuhua HaoSavvas ChristoforidisFujun QinSavvas LampridisFumiaki UchiumiSawsan A. ZaitoneFumitaka KogaSayantani ChowdhuryFuyi WangSayantani Sarkar BhattacharyaFuyuki SatoSazan RasulG. M. LanzaScott H. OlejniczakGábor BaloghScott I. SimonGábor HalmosScott McCombGábor JancsóSe Kyoo JeongGábor L. KovácsSean Chun Chang ChenGábor Nagy-GróczSebastian D. SchaeferGábor TernákSebastian Demyda PeyrásGábor VargaSebastian FindekleeGabriel Costa A. Da HoraSebastian GnatGabriel HancuSebastian KalamajskiGabriel SantpereSebastian M. StrauchGabriel WajnbergSebastian SchnaubeltGabriela Dumitrita StanciuSebastian SchwamingerGabriela HrckovaSebastian WawrockiGabriela LootsSebastian YuGabriele CarulloSebastiano Alfio TorrisiGabriele CeccarelliSebastiano CiccoGabriele GrunigSébastien WälchliGabriella CsíkSeddik HammadGabriella HorváthSeema DangwalGabriella ZitoSeema GuptaGaetano GalloSeema SinghGaetano SantulliSeifedine KadryGagan D. FloraSeil KimGaganpreet KaurSeila Lorenzo-HerreroGalina KochnevaSeisuke OkazawaGalina KoltsovaSelami DemirciGamaleldin OsmanSelestina GorgievaGang PeiSelim KuçiGannon C. K. MakSelvakumar SubbianGarcía-Moreno Nisa FranciscaSemon WuGareth MorganSenthilkumar SankararamanGargi MahapatraSeong Soo A. AnGarikoitz AzkonaSeong-Hee Maria KoGary FirestoneSeongjae JangGary McLeanSepanta HosseinpourGaspare PalaiaSerena De SantisGatikrushna SinghSerena DuchiGautam PareekSerena MilanoGautam SethiSerena PellegattaGawęcki MaciejSerena Y. TanGaylen L. EdwardsSerge NatafGeir BjørklundSergei G. GaidinGelu OnoseSergei KirischukGema FrühbeckSergej PirkmajerGema Martinez-NavarreteSergey BolevichGemma ArsequellSergey KiselevGemma PalazzoloSergey KolomeichukGeng-Shi JengSergey KulemzinGennadiy A. KostinSérgia VelhoGennady ChuevSergio FacchiniGentaro IzumiSergio FrutosGeoffrey C. LiSergio Martínez-HervásGeorg GelbeneggerSergio OcchipintiGeorge A. PapakostasSergio Rius-PérezGeorge A. TavartkiladzeSerhii HolotaGeorge BaylissSeri JeongGeorge Calin DindeleganSerra MarcelloGeorge Eric BlairSestina FalconeGeorge Mihai NitulescuSeth CoreyGeorge NotasSeth FrietzeGeorge P. ParaskevasSeth PincusGeorges LeftheriotisSeverino PersechinoGeorges VassauxSevinci PopGeorgeta Violeta TruscaSeyed Alireza R. SalamiGeorgia KarpathiouSeyed Khosrow TayebatiGeorgiana Smaranda MartisShadab MdGeorgios GaitanisShahab UddinGeorgios GalaziosShahanshah KhanGeorgios KatsarasShahid Ali KhanGeorgios S. MarkopoulosShahin HomaeigoharGerald CohenShahnawaz AnwerGerald Fritz SchusserShailendra Pratap SinghGerald W. FeigensonShailesh KumarGerardo Daniel FidelioShalini K. SharmaGerardo MusuracaShamir CassimGerardo PrietoShamroop Kumar MallelaGerd Faxén-IrvingShams TabrezGergely GyimesiShamsudheen VellarikkalGerman Dominguez VíasShan ZhengGertjan Jan L. KaspersShangrong FanGhais KharmandaShangru LyuGheorghe IliaShankar Jaikishan EvaniGheun-Ho KimShanlin KeGholamali RahnavardShaoan WangGiacinto BagettaShao-Bin WangGiacomo BaimaShao-Chen LeeGiacomo CavallaroShao-Jun TangGiacomo FarìShaopeng PeiGiacomo MiserocchiShao-Ting Jerry TsangGiada AmodeoShao-Wen HungGiada MagniShaoxun WangGiampiero NeriShaoyong LuGian Maria BusettoSharat ChandraGiandomenico AmicoSharmila FagooneeGianfranco AlpiniSharmila GhoshGianfranco NataleShashikumar SalgarGianfranco SantovitoShaul SchreiberGianina DodiShaw-Ji ChenGianluca AguiariShazia BanoGianLuca ColussiShebli AtrashGianluca EspositoSheida MoghadamradGianluca GaidanoSheikh Mohammad Fazle AkbarGianluca GennarelliSheikh RiazuddinGianluca M. TartagliaSheketha R. HauserGianLuca MoronciniShekhar SahaGianluca NazzaroSheng Dean LuoGianluca SferrazzaSheng ZhangGianluigi CalifanoSheng-Hung ChenGianluigi MazzoccoliShengkun XieGianluigi PastaShengshuai ShanGianmarco AbbadessaSherry MansourGianmarco PallaviciniSherry S. CollawnGianna CamiciottoliShi Song RongGianni Di GiorgioShi YaoGianpaolo PapaccioShian-Ren LinGianpiero ZamperinShibei WuGiau VoShibi LikhiteGilbert GallardoShichen ShenGilberto A. GonzalezShicheng GuoGilles LemaîtreShigeki MatsubaraGilles LuijtelaarShigeki SugawaraGina GheorgheShigeru MatsumuraGina-Cecilia PistolShigeto TakebayashiGino GrifoniShihlung ChenGioele CastelliShih-Min HsiaGiordano PeriniShiho NaitoGiorgi KuchukhidzeShihori TanabeGiorgia AilunoShih-Yen LoGiorgio ArcaraShin EnosawaGiorgio FacchettiShin TakasawaGiorgio FilosaShin-Cheh ChenGiorgio FuianoShindu ThomasGiorgio LofreseShing-Hwa LiuGiorgio ManginoShinichi KinamiGiou-Teng YiangShin-ichi NakakitaGiovanna CalabreseShinje MoonGiovanna CaparelloShinji TakamatsuGiovanna FerraioliShinn-Jyh DingGiovanna MantiniShinpei OgawaGiovanna PannuzzoShintaro HorieGiovanna RicciShintaro SukegawaGiovanni BarillariShinya HasegawaGiovanni BellomoShinya MatsuzakiGiovanni BrunoShiqiang GaoGiovanni CrisafulliShirley D’SaGiovanni CugliariShiv Dutt PurohitGiovanni Francesco MarangiShiv Prakash VermaGiovanni GaleotoShiv Shankar VermaGiovanni L. BerettaShiva Theja Reddy SomaGiovanni N. RovielloShiwani SharmaGiovanni PalleschiShiwei ZhuGiovanni PaolinoShizuka UchidaGiovanni PesShobini JayaramanGiovanni RaugeiShofiul AzamGiovanni RollaShogo HamaguchiGiovanni ScarzelloShowkat DarGiovanni TarantinoShreesh Kumar OjhaGiovanni TossettaShrikanth GadadGirish PattappaShriram VenkatesanGisella VischiniShuai WangGiulia AbateShuan-Pei LinGiulia DonadelShu-Fen ChuangGiulia FerrarazzoShugo SuzukiGiulia PignataroShuji MomoseGiulia SciosciaShuji OzakiGiuliano RamadoriShujiro HayashiGiulio Francesco RomitiShu-Jui KuoGiuseppe A. PalumboShu-Ling TzengGiuseppe AmbrosioShuncong WangGiuseppe AmorusoShunqing LiangGiuseppe BarlettaShu-Pin HuangGiuseppe BiaginiShuqiang WangGiuseppe BiscagliaShwu-Chen TsayGiuseppe BroggiShyam Kumar GudeyGiuseppe Carlo IorioShyam MoreGiuseppe CirilloSi ChenGiuseppe FanciulliSibhghatulla ShaikhGiuseppe FerrandinoSiddharth SunilkumarGiuseppe GulloSilvana AlfeiGiuseppe LanzaSilvano DragonieriGiuseppe LiberatoreSilvia BarbonGiuseppe LosurdoSilvia BistiGiuseppe MaruccioSilvia CorrochanoGiuseppe MasciaSilvia Di AgostinoGiuseppe MaulucciSilvia DottiGiuseppe MiserocchiSilvia GalbiatiGiuseppe MurdacaSilvia M. ArribasGiuseppe PassarinoSilvia MarracciGiuseppe RivaSilvia MonticoneGiuseppe SarpietroSilvia PalmaGiuseppe SciamannaSilvia SauledaGiuseppina CatanzaroSilvia SelleriGiuseppina LaganàSilvia SvegliatiGiuseppina MalcangiSilvia ToricesGiuseppina NoccaSilvia VanniGiuseppina PalladiniSilvia VidalGladys KoSilvio HemmiGloria Álvarez-SolaSilvio IontaGloria ManzottiSilvio VeigaGloria Olaso-GonzalezSimin SharifiGobinath ShanmugamSimon Chi Chin ShiuGohar FakhfouriSimon FunnellGonçalo J. JustinoSimon HeilbronnerGoorak KwonSimona BungauGoran SedmakSimona CandianiGordana Blagojević ZagoracSimona Delia NicoaraGordana Wozniak-KnoppSimona Delle MonacheGöte SwedbergSimona IftimieGoto YoshikazuSimona PergolizziGouveia Zelia LiciniaSimona RuțăGraça SoveralSimone BattagliaGrady NelsonSimone BeninatiGrasiele SausenSimone Ciofi BaffoniGrazia PalombaSimone FioriGraziella TuratoSimone GalloGrażyna BudrynSimone MorselliGrazyna NiewiadomskaSimone PatergnaniGrażyna NowickaSimone SforzaGreg Neil BowdenSimone WajnerGregory CaputoSimonetta D’ErcoleGregory J. TsaySimons SvirskisGreta FaccioSimote FoliakiGrzegorz GrześkSimranjeet KaurGrzegorz PorebskiSinisa DjurasevicGrzegorz SumaraSiqi GuoGrzegorz ZagułaSiqi HuGuangda PengSiu Hong Dexter WongGuanghui MaSivarajan KumarasamyGuangzhao LiSiwei ZhangGuan-Jhong HuangSjoerd T. SchettersGudjon Elvar TheodorssonSladjan Z. PavlovićGuendalina ZuccariSławomir GonkowskiGuglielmo ManticaSławomir KujawskiGuido ZavattaSławomir WołczyńskiGuillaume FichesSławomir ZmonarskiGuillaume GiraudSławomira Drzymala-CzyzGuillermo López LluchSmijin K. SomanGunnar RonquistSmita NairGünter LorenzSmrithi PadmakumarGuoping ZhengSmriti SinghGuoqing QianSneha RatnapriyaGuorong LiSnehal ShuklaGuowei KimSocrates DokosGuozheng WangSofia GuedesGurdeep MarwarhaSofia ParrasiaGurinder SinghSofia VasilakakiGurpreet Kaur AroraSohini ChakrabortyGustavo Alberto ChiabrandoSoléakhéna KenGuy RostokerSom DevGuya MarconiSomaira NowsheenGwen M. ClarkeSomchai ChutipongtanateGyörgy BallaSonali NashineGyórgy ParaghSonam MittalGyörgy Tibor BaloghSoni DhruvKumarGyörgy TrencsényiSonia Benítez GonzálezGyörgyi MűzesSónia C. CorreiaHaewon ByeonSonia LaneriHae-Won KimSonia SirisiHahn Young KimSonika TyagiHaidong FengSonu M. M. BhaskarHaifeng YangSonu SinghHaiyan YangSoo Young LeeHaiyang CuiSoochong KimHaizhen Andrew ZhongSoon-Chan KwonHajeewaka C. MendisSooyeon LeeHakan AldskogiusSoraya L. VallesHalilibrahim CiftciSoren U. NielsenHan Siong TohSorin HostiucHana HansikovaSorina DinescuHanan Hassan Abd-ElhafeezSoumen SahaHanefí ÖzbekSouvick RoyHanna Augustyniak-BartosikSowjanya ThatikondaHann-Chorng KuoSperanza RubattuHanneke Kwakkel-van ErpSpiros VlahopoulosHanno M. WitteSpyridon KintziosHans De LoofSpyros PapageorgiouHans G. DrexlerSreenivasulu KilariHans Georg SchaibleSrikanthan RameshHans KnechtSrinivasa SubramaniamHans-Christian SiebertSriram SundaravelHans-Gert BernsteinSrjit DasHans-Joachim SchulzeStamatia TheodoridouHan-Soo KimStamatoula TsikrikaHanumantha Rao MadalaStanislav KotlyarovHany I. SakrStanislava PankratovaHanying ChenStanley ChanHao NieStavros TryfonHao-Jui WengStefan BurdachHarald KrentelStefan D. MomčilovićHarald MischakStefan MihaicutaHardeep Singh TuliStefan TanglHardy RideoutStefania BrozzettiHari Krishna NamballaStefania CantoreHarikanth VenkannagariStefania MarcuzzoHarikumar RajaguruStefania MerighiHarini MohanramStefania ScaliseHarold Y. LauStefania SciallaHaroon KhanStefanie SchirmeierHarpreet KaurStefanie WillekensHartmut KühnStefano BellostaHaruhisa KikuchiStefano BellucciHaruki KoikeStefano BonassiHaruki UsudaStefano CanosaHaruto NishidaStefano CordioHåvard J. HaugenStefano DastoliHayami ShinyaStefano De ServiHayato TadaStefano LuinHayato TsukamotoStefano LuzzagoHe ZhuStefano SotgiuHeather BensonStefano TuriHeather EnrightStefano VeraldiHeather WilkinsSteffen JunkerHector M. Mora-MontesStela McLachlanHee Kyoung JooStelios LoukidesHee-Chul AhnStephan BeiskenHei KimStephan DreschersHeiko SorgStephan LauxmannHelder MarcalStéphan TroyanovHelen IrvingStéphane ChavanasHelena MartynowiczStephane R. GrossHelena P. FelgueirasStephanie DauthHélène BoeufStephanie DuguezHélène CôtéStéphanie LefebvreHélène LemieuxStephanie MangesiusHelene SpeyerStephanie MenikouHéloïse BourienStephanie RoesslerHenning FenselauStephanie T. YerkovichHenrich FrielinghausStephanie WattsHenrik LaurellStephen AdelsteinHenrike FischerStephen J. RichardsHenry NordingStephen J. TerryHenry Puerta-GuardoStephen J. WalkerHenry SutantoSteve CarlanHerbert Ryan MariniSteve ShnyderHermann Von GrafensteinSteven D. ColquhounHerminia Gonzalez-NavarroSteven EsworthyHermona SoreqSteven HeatonHernan Diego FolcoSteven MutsaersHerwig CerwenkaSteven WillowsHesham El-EnshasySubhadeep ChakrabartiHesham El-SeediSubhadip ChoudhuriHidehiko KikuchiSubhasis ChattopadhyayHideki SugiiSubir Kumar JuinHideo KatoSu-Boon YongHidetoshi TsudaSubramanyam DasariHideyuki KinoshitaSubrat BhattamisraHideyuki MaedaSubrata DebHilda E. GhadiehSuchismita RahaHilda Petrs-SilvaSudhir KshirsagarHimanshu KathuriaSudhirkumar YanpallewarHinrich P. HansenSueli Coelho Da Silva CarneiroHipolito NzwaloSuhrid BanskotaHiro SatoSui MaiHiroaki NakagawaSukhbir KaurHiroaki WakimotoSuman AlishettyHirofumi JonoSuman ChowdhuryHirofumi NakatomiSuneel KumarHirokazu SakamotoSung Hoon LeeHiroki MiuraSung-Kun KimHiroki ToyodaSung-Lang ChenHiroki YokotaSung-Whan KimHiromi SakaiSung-Wuk JangHiromichi YumotoSunil S. AdavHironori YoshinoSunil SinghHiroshi HandaSun-Joon MinHiroshi KurokiSunny S. K. ChanHiroshi MiyamotoSupandeep Singh HallanHiroshi TakemoriSupriya ChakrabortyHiroshi YoshidaSuresh Ponnayyan SulochanaHiroya YamadaSuresh ThanguduHiroyuki TsuchiyaSuresh VeeramaniHiroyuki UetakeSusan ChristoHiroyuki WakiguchiSusan RichterHiroyuki YamadaSusana ArmestoHitendra Singh SolankiSusana B. BravoHo TsoiSusana CardosoHolger Hans Herman ErbSusana SilvaHolly A. RoySusanna KällbomHong Jin KimSushil Kumar ShakyawarHong Sheng ChengSusumu SuzukiHong Yong PehSusumu TanakaHong ZanSvend LindenbergHongfei ZhaoSvetla TodinovaHonghe LiuSvetlana TamkovichHongping WeiSvetlana V. GuryanovaHongyang XuSvetoslav TodorovHooi-Leng SerSwagata Ghatak GhatakHoon KimSwaroop Kumar PandeyHoon-Ki SungSwayam Prakash SrivastavaHoria HaragusSweta B. PatelHoria OprisSybille HasseHoria PăunescuSyed A. A. RizviHossam M. HassanSyed Farhan AhmadHrvoje RimacSyed Furqan QadriHsien-Bin HuangSyed Husain Mustafa RizviHsien-Yuan LaneSyed Sayeed AhmadHsin-An ChangSygkliti-Henrietta PelidouHsin-Yuan ChenSylvain FraineauHsiu-Mei ChiangSylwia Dzięgielewska-GęsiakHsiuying WangSzymon JanczarHsiyuan HuangSzymon MalinowskiHsueh-Wei ChangT. S. AnilkumarHsuheng YenTabito KinoHu HuangTadakazu KondoHuachun CuiTadashi FuruyamaHuafeng WangTadashi ItoHuarong ChenTadayoshi KagiyaHuaxin ShengTadeja KuretHueng-Chuen FanTadeusz PawelczykHuey-Shan HungTahmina Nasrin PolyHugo Arasanz EstebanTajana FilipecHugo FragaTakaaki HirotsuHui LiTakaaki SenbonmatsuHui LiuTakahiro HasebeHui WangTakahiro InoueHuibin LvTakahiro KannoHui-Hua HsiaoTakahiro MasudaHui-Yen ChuangTakahisa GonoHui-Yun ChengTakahisa KanekiyoHumbert González-DíazTakako OsakiHumberto Sanchez GonzálezTakanori KuboHung-Chi ChengTakao IshikawaHung-Chuan PanTakashi AguiHung-Jen WangTakashi HimotoHung-Pin TuTakashi KudoHung-Yin LinTakashi MatsushimaHung-Yu HuangTakashi NomizoHuub SchellekensTakashi SaitoHwajin KimTakashi UebansoHye Jin YouTakashi YoshiyamaHyemin KimTakayuki MasakiHyeongwon YuTakayuki NakagomiHyeonji KimTakayuki OhkuriHyonchol JangTakemi OtsukiHyun Jin KwunTakemichi FukasawaHyun Sook HongTakeshi AdachiHyun Soon GweonTakeshi KajiharaHyunju LeeTakeshi KawasakiIacopo GesmundoTakeshi KikuchiIain HargreavesTaketo YamadIbrahim Fatih CengizTakuji TanakaIbrahim M. SayedTakuma InagawaIbtisam TothillTakumi IshizukaI-Chien WuTakumi TakeuchiIda AringerTakuya KoieIda CariatiTakuya TadaIda PastoreTal DvirIgea D’AgnanoTally Naveh-ManyIgnacio HernandezTamal SadhukhanIgnazio Gaspare VetranoTamara ČačevIgor DumicTamara Fernández CabadaIgor JakovcevskiTamara G. AmstislavskayaIgor KhatkovTamara GulicIgor KukhtevichTamás DócziIgor P. KaidashevTamás JuhászI-Jung TsaiTamas LetohaIlan MerdlerTamás OllmannIlaria BortoneTamer S. KaoudIlaria MormileTammy KindelIlaria PelusoTang LiIlaria TamagnoTania ColasantiIlavenil SoundharrajanTania Quesada-LópezIldiko HorvathTânia S. MoraisIldiko SzantoTanja Grubić KezeleIlia StamblerTanja ZidaričIlya UlasovTanya MiuraImmacolata PietraforteTao HuangImtiyaz Ahmad WaniTao LiIn-Ah LeeTao ZhangIndu G. RajapakshaTaobo HuInes Bilic-ĆurčićTapas K. MandalIngrid CipakovaTareq SalehIngrid PrkačinTatiana AstrelinaInna P. GladyshevaTatiana N. BorodinaInna SolomonovTatiana V. VygodinaInnokentii E. VishnyakovTatiana Yu KuznetsovaInyeong ChoiTatijana ZemunikIoan SporeaTatjana PirmanIoana CabaTatjana RadosavljevićIoana Dorina VlaicuTatsuji MizukamiIoana MozosTatsuo KandaIoana StreațăTatsuo KawaiIoana-Mihaiela CiucaTatsuo ShiodaIoanina ParlatescuTatsuya AbéIoanna NikitopoulouTatsuya KobayashiIoannis CharalampopoulosTatyana O. PleshakovaIoannis GiantsisTatyana VlaykovaIoannis NikolakakisTavarekere N. NagarajaIoannis PaterasTe-Chun ShenIoannis PetrakisTe-Jen SuIoannis ToumpoulisTeodora Alexa-StratulatIoannis VamvakarisTeodora TelecanIoannis ZachosTeodor-Doru BrumeanuIonela NicaTeodorico De Castro RamalhoIonut LuchianTeresa AlmeidaIonut TudoranceaTeresa Carbonell CamósIosif MarincuTeresa GrzelakIqram HussainTeresa Rubio-TomásIraia Muñoa HoyosTeresa RussoIraide AllozaTereza GabelićIram MurtazaTereze KatzensteinIrena Brčić KaračonjiTerry LichtorIrena MaliszewskaTeruhiko ImamuraIrena MladenovaTeruyoshi TanakaIrena TabainTessho MaruyamaIrene CarusoTetsufumi TakahashiIrene KarampelaTetsuya IkedaIrene Santos-BarriopedroTetsuya OgawaIrene ScaleraThemis AlissafiIreneusz SowaTheo KrausIrfan A. RatherTheocharis G. KonstantinidisIrfan KhanTheodora PapamitsouIria Seoane-ViañoTheodore CumminsIrina Georgiana MocanuTheodoros AndroutsakosIrina IelciuTheodoros EleftheriadisIrina M. Le-DeygenTheodoros KalampokasIrina MateiTheofilos M. KolettisIrina MukhinaThi Thao Linh NguyenIrina NegutThierry HauetIrina St. LouisThomas A. HorbettIrina V. KiselevaThomas BiedermannIrina-Georgeta SufaruThomas E. SerenaIrina-Iuliana CostacheThomas FinkIris Maria ForteThomas GilmoreIris Xiaoxue YinThomas GreitherIrmgard TegederThomas HankIrshad Ahmed HajamThomas KeilIruthaya Pandi Selestin RajaThomas KinneyIrzal HadžibegovićThomas LiehrIsaac BragaThomas LutzIsabel ConceiçãoThomas M. FreimanIsabel Legaz PérezThomas MüllerIsabel MarzoThomas MürdterIsabella ParoliniThomas StammingerIsabella SalzerThomas TiefenboeckIsabella ZironiThomas W. BeckerIsabelle Galy-FaurouxThorsten BraunIsaiah ArkinTianyu LiIsao NagaokaTiberiu Paul NeaguI-Shiang TzengTiina M. AndersenIsmaheel O. LawalTim RobertsIsmail FidanTim WalkerIsmail YenerTimo Z. Nazari-ShaftiIsmini E. PapageorgiouTimofei ZatsepinIssam KoleilatTimoteo MarchiniIssan ZhangTimothy KeifferIta HadžisejdićTimothy VeenstraI-Ta LeeTina McKayItzhak FischerTina Tičinović KurirIulia-Ioana Stanescu-SpinuTing HanIurii S. StafeevTingxiang YanIva LakićTingyu ChangIvan BrandslundTinhinane FaliIván CarreraTiziana BachettiIvan Cruz-ChamorroTiziana FeolaIván HenríquezTiziana GreggiIvan SrejovicTiziana MargariaIvan StoikovTiziana RaiaIvana AntonucciTiziano VerriIvana JochmanováTobias EttlIvana KacirovaTobias OsterlundIvana KraljevicTobias StraubIvana SamarzijaTom DariusIvana ŠkrlecTom L. BroderickIvette Buendía RoldánTomas BlancoIwona Ben-SkowronekTomas FiedlerIwona Grabowska-KowalikTomáš HavránekIwona SzydłowskaTomas SimurdaIwona WertelTomasz BlicharskiIzabela Korona-GłowniakTomasz BuchwaldIzabela Małysz-CymborskaTomasz FrączykIzabela Sadowska-BartoszTomasz J. PrószyńskiIzabela ZakrockaTomasz KluzIzildinha MaestáTomasz KoziorJ. Francis BorgioTomasz KubrakJ. S. Hill GastonTomasz PodgórskiJacek MalejczykTomasz ŚledzińskiJacek NyczTomasz StompórJacek PiatekTomasz SzczepańskiJacek SzepietowskiTomasz TokarekJacinta SimmonsTomasz W. KaminskiJack J. W. A. Van LoonTomasz WolnyJacopo Junio Valerio BrancaTomiki SumiyoshiJacqueline CloosTomislav BokunJader de OliveiraTomislav TostiJadwiga ManiewskaTommaso CaiJae Boum YoumTomohiko SadaokaJae Ho LeeTomohiro MatsuoJae Min ChoTomohiro MizobataJaeho ChungTomohiro YazawaJae-Ho LeeTomokazu ShimizuJae-Hyung ParkTomomi NakaharaJaejoon LimTomoyuki IwataJae-Seok KimTomoyuki KawaseJa-Eun KimTomoyuki MaruoJagadheshwar BalanTong WangJahnavi AluriTong-Hong WangJaison JeevanandamTony RodriguesJake Jianjun WenTorsten LowinJake WhangToru TaguchiJakob TroppmairToshihiko MatsumotoJakub GrzesiakToshihiko TashimaJakub KornackiToshihiro KushibikiJakub MatusiakToshikazu SuzukiJakub NalepaToshinori ShimanouchiJakub RokToshio HattoriJakub SuchánekToshiro AraiJakub ZdartaToshiyuki MinamiJames ChenToti LucaJames ChowTovit RosenzweigJames D. BrooksToyoaki OhbuchiJames LaffertyToyonori TsuzukiJames LewisTraian DumitrașcuJames M. GruschusTrang NguyenJames P. BennettTravis B. NielsenJames WalterTravis E. GrotzJamir Pitton RissardoTravis P. SchrankJan BakosTrevor ArcherJan BilskiTriantafyllos DidangelosJan ChojnackiTrim LajqiJan Czesław KotarskiTrine L. Toft-BertelsenJan EggerTruls E. Bjerklund JohansenJan HagemannTse-Ying LiuJan KorbeckiTsung-Jen WangJán LehotskýTsung-Jung LiangJan MieszkowskiTsung-Rong KuoJan PithaTsuyoshi OhtaJan StȩpniakTsvetelina BatsalovaJan TrnaTsvetelina VelikovaJan Walter SchroederTuan L. PhanJana FrankováTuba Çiğdem OğuzoğluJana Sopková-De Oliveira SantosTuli MukhopadhyayJanaki IyerTunde CsepanyJanani ParameswaranTursonjan TokayJane K. NguyenTuru GáborJane LiesveldTusty-Juan HsiehJane WelshTvrtko HudolinJanez MravljakTyson GraberJanin ReifenrathTze-Kiong ErJanja TrcekTzong-Shyuan LeeJanka BábíčkováTzung-Yan LeeJanna G. KoppeTzu-Ping KoJanovec VaclavTzu-Yen HuangJanusz KsiazykUdaiyappan JanakiramanJanusz SolskiUgo CarraroJara Pérez-JiménezUgolino LiviJaromir AstlUjjal Kumar BhawalJaromír MyslivečekUlises De La Cruz-MossoJarosław CzyżUlrich SalzerJarosław J. SakUlrika Von DöbelnJaroslaw KierkusUmar HayatJarosław PaluszczakUmberto AnceschiJarosław PinkasUmberto BasileJaroslaw ZmudzkiUmberto GaragiolaJasenka WagnerUmberto MalapelleJasjit S. SuriUri AsheryJasna KusturicaUrszula KarczmarczykJason J. WangUte RömlingJason P. SchwansUtkarsh H. AcharyaJason S. BuhrmanVadanasundari VedarethinamJason Tze Cheng TzenVadim B. KrylovJaved AhmadVahideh RabaniJavier C. AnguloValentin Nicolae VarlasJavier Caballero-VillarrasoValentina BudaJavier EnrioneValentina CastelliJavier Fernández-RuizValentina CitiJavier MartínValentina CossigaJavier Marti-RujasValentina Di NisioJavier TamayoValentina GinevičienėJawhar GharbiValentina LatinaJaw-Yuan WangValentina PileczkiJay TrivediValentina RubinoJayachandran VenkatesanValentina SancisiJayakanthan KabeerdossValentina VelleccoJayne MurrayValeria PanzettaJean Louis VincentValeria PasciuJean MukherjeeValeria SogosJean VanderpasValeria ZampiniJean-Baptiste Le PichonValerian DormoyJean-Denis TroadecValerie Diane ValerianoJean-François PeyronValerie E. RymanJean-Marc BarretValerio CicconeJean-Marie ExbrayatValerio IebbaJean-Marie RuysschaertValerio LeoniJeanny B. Aragon-ChingValerio VolianiJean-Philippe PinValery V. LikhvantsevJean-Pierre GagnerVamsee MallajosyulaJean-Yves PiergaVance G. NielsenJeena BansalVanessa GilJeevithan ElangoVanessa PinhoJeffrey ChanVasia S. MavrogianniJeffrey KnaufVasil D. SimeonovJeffrey L. MoranVasileios SiokasJeffrey WangVasileiou PanagiotisJegan IyyathuraiVasilios PergialiotisJelena RoganovicVasily Nikolaevich SukhorukovJelica Grujić-MilanovićVassilios SikavitsasJen-Chang YangVassilis DourtoglouJenchih TsengVassiliy TsytsarevJennifer CaseyVasudev BallalJennifer DowlingVasudeva IyerJennifer FangVedat TopsakalJennifer MytychVeena SangkhaeJennifer PattersonVeera Ganesh YerraJennifer VandoorenVeeranoot NissapatornJens HahneVelia D’AgataJens SchusterVeronica Alexandra AntipovaJeong DongtakVeronica MarrellaJeong-An GimVerónica Moreno-ViedmaJeong-hae ChoiVeronica SansoniJer-Ming ChangVeronica SteriJérôme AddaVeronika HuntosovaJérôme EeckhouteViacheslav NikolaevJérôme PiquereauVibeke BratsethJerónimo González-BernalVibor MilunovićJerzy ChudekVictor AtuchinJerzy JuśkiewiczVictor E. NavaJes-Niels BoeckelVictor Ivanovich SeledtsovJesse ArbuckleVíctor M. RiveraJessica Da Gama DuarteVictor MatheuJessica Dal ColVictor RevinJessica Maria AbbateVictor van BeusechemJessica RosatiVictoria MacBeanJesús Adolfo García-SáinzVid MatišićJesús BalsindeViet-Khoa Tran-NguyenJesús DevesaVijay BoggaramJesus M. Martin-CamposVijay ReddyJesús PalomeroVijeta SharmaJesus S. JimenezVikas MalojiraoJesus VelaVikas SinghJezabel BlancoVikas YadavJi Hoon JungVikrant RaiJi Wan ParkViktor DreminJiabing FanViktor KokhanJiake XuViktor ReshetnikovJiale YangViktoria JeneyJialei DuanVilma RatautaitėJiali YuVinay KumarJia-Ling RuanVinay MandatiJian XuVinayak JoshiJian YinVincent CalvezJianfeng HuangVincent CastelainJiang LiuVincent PonsJiangning YangVincent Van DuinenJianlei GuVincent YeungJianling XieVincenza GianfrediJiann-Jou YangVincenzo BrancaleoneJiann-Ruey HongVincenzo CastiglioneJianqiang XuVincenzo De FilippisJianxiang XueVincenzo Di StefanoJianxin WangVincenzo FiorentinoJianxuan WuVincenzo Nicola TalesaJiaqi ZhangVincenzo NuzziJiaquan YuVincenzo QuagliarielloJiawei WangVincenzo SorrentinoJie ZhangVinita BharatJie ZhuVinod V. T. PadilJien Jiun ChenVinod VijayakurupJihan XiaVinodh KakkasseryJihane Homman-LudiyeVioleta G. TruscaJihwan HaViorica PatruleaJih-Yang KoVirag VasJijun HuangVirginia BrancatoJimin GuoVirginia TancrediJin BaiVirginie Monnet-CortiJin Hyuck ParkVishal K. ChavdaJin Seok LeeVishal RawjiJin ZhangVishal Vishnu TewariJinan LiVita GolubovskayaJinchul KimVitalie LisnicJindrich KopeckýVito TerlizziJing ChenVítor CavadasJing PangVitor ParolaJing’an LiVittoria Di MauroJingjing WuVittorio CalabreseJingrui ChenVivek K. KashyapJingxia LiuViviana OnofreiJingyi ChiViviana VallacchiJin-Hong ShinVlad Constantin TofanJinli WangVlad PorumbJinming HanVladimir A. LazarenkoJinn-Rung KuoVladimir A. ShvartzJinsong HaoVladimir GureevJinsoo SeoVladimir I. KalininJinwei ZhangVladimir LupashinJiranuwat SapudomVladimir PisarevJiri KassaVladimir SobolevJiro KishimotoVladimir VladimirovJishnu KrishnanVladislava GusarJisu LiVlasta BariJitendra KanaujiyaVolkan ArisanJiunn-Horng KangVolkert A. L. HuurmanJiun-Wen GuoVrinda GoteJiyang CaiVyacheslav RyabovJiyeon HamVyron GorgogietasJoan BagóWaldemar WagnerJoan C. KrepinskyWaleed Bakry SuleimanJoan Carles Escolà-GilWalther BildJoana BarbosaWan-Chi TsaiJoana CaetanoWanda MączkaJoana DamásioWang FangJoana GonçalvesWang TaoJoana VitalléWan-Jin ChenJoanna BartosińskaWanting HanJoanna BialekWaqas NawazJoanna CzwartosWayne CarterJoanna DobrzańskaWei ChenJoanna FilipowskaWei FeiJoanna ListosWei GuJoanna StefanowiczWei LinJoanna SuliburskaWei Long NgJoanna SzczepanekWei ShaoJoão Augusto FerreiraWei Shiang LinJoão Carlos SilvaWei WangJoão F. ManoWei ZhangJoão Miguel Marques dos SantosWei ZhongJoao R. GomesWei ZhouJoão Ramalho-SantosWei-Chieh LeeJoaquim CarrerasWei-Chih ChenJoaquim OristrellWeifeng ZengJoaquín María CamposWei-Hsin SunJobst LandgrebeWei-hsiung YangJochen MaurerWeihua DingJochen PölingWeikun XiaoJoerg LindenmannWeiran ShanJohan HoebekeWeirong XingJohann EberhartWei-Sheng ChenJohanna CastañoWei-Sheng LinJohanne PoissonWeiwei HanJohannes BoltzeWeiyi XuJohannes Frank Wilhelmus NijsenWeiyu LiJohannes HoferWeiyun ShiJohannes LeiererWen ChenJohannes SchulzeWen ShiJohannes StoecklWen WangJohannes StraussWen-Chin LeeJohn A. St. CyrWen-Der WangJohn AshtonWen-Hao YangJohn BennettWen-Sen LeeJohn BrachtWen-Tsong HsiehJohn D. PapatriantafyllouWenxin ZhengJohn GreenWenyu WangJohn HealeyWesley SchaalJohn Lalith Charles RichardWiesław KacaJohn M. PerryWieslawa Agnieszka FogelJohn Man Tak ChuWilfried PoschJohn ManionWilliam B. GrantJohn N. PlevrisWilliam E. BenitzJohn O. OnukwuforWilliam RassmanJohn PresleyWilliam SchwanJohn PufferWilliam T. Gunning IIIJohn S. MitchellWilliam T. HellerJohn StanleyWilliam Ted BrownJohn W. SteeleWillibald WonischJoke De PauwWim CalameJolanta DorszewskaWindy McNerneyJolanta Nawrocka-RutkowskaWito RichterJolanta PolakWładysław LasońJolanta RzymowskaWojciech KalasJolanta VaskelyteWojciech PaslawskiJonas CicenasWojciech SmułekJonas WixnerWolfram GronwaldJonathan A. LindquistWolfram H. GerlichJonathan Barroso-GonzálezWolfram S. KunzJonathan G. AchesonWonchung LimJonathan GrasmanWon-Ha LeeJonathan GreenbergWoojin KangJonathan LuisiWoojin KimJonathan M. HarrisWoon-Man KungJonathan R. DimmockWooyeong ParkJonathan ReichnerWorawit LouthrenooJonathan Robert WeitzWu Chih-LungJonathan RoydsWu QiuJonathan S. ReichnerWujie ZhangJong Kook ParkWupeng LiaoJonghoe ByunWylie BenjaminJong-Koo KimXanthopoulos KonstantinosJongsub MoonXavier Gallart-PalauJoni H. YlostaloXavier X. Sastre-GarauJooMan ParkXenia FilipJoon Hyuk ChoiXesús FeásJoon W. ShimXi LouJoost KluiverXia WeizhongJoost R. van der VorstXiang WangJörg KleeffXiang-Ping ChuJorge A. GuimarãesXiangyu RenJorge CervantesXiangyu ZhangJorge del Rosario Fernández SantosXiaochen HeJorge FeitoXiao-Dong ChenJorge GalvezXiaofei LiJorge GonçalvesXiaofeng Allen SuJorge Jorge SimonXiaofeng QiJorge N. R. MartinsXiaojie LiuJorge SimonXiaokuang MaJos M. B. M. Van Der VossenXiaoli YuJosé A. CarriónXiaoping PuJosé A. MartinsXiaoshu XuJosè Ángel FontenlaXiaosu (Frank) HuJose Antonio Cañas MañasXiaotang MaJosé Antonio LupiáñezXiaoting HongJosé Antonio Morales-GonzálezXiaoyu SongJosé Bernardo Noronha MatosXiaxia DiJosé Bustos-ArriagaXie BingJosé C. S. dos SantosXin ChenJose Caparros-MartinXin CuiJosé ChiesXin LiJose Felix Moruno ManchonXin SunJose IglesiasXin XingJosé López-CastroXin ZhangJosé Luis FerranXingchen PengJosé Luis Pérez-CastrillónXingchun GaoJosé Luis ReyesXinglei LiuJosé MontesXinli LiJosé Pedraza ChaverriXinxing ZhangJose PerdomoXinyi SuJose Sanchez-AlcazarXisha WangJose TudelaXuan MeiJosé-Antonio Girón-GonzálezXudong CaoJosef HalamekXuehua XuJosef SmolleXuejuan JiangJosef ZichaXun SongJosep JulveYafeng LiJosep Lluís Clua-EspunyYair LópezJosep M. Cambra BortYajuan SuJoseph KwongYan JinJoseph MoxonYan LiJoseph NassifYan SunJoseph PapamatheakisYan V. ZubavichusJoseph PierreYan YangJosé-Tomás NavarroYanan LiuJoshua BarzilayYang CaoJoshua C. DoloffYang HeJosip Andelo BorovacYang JiangJosip DelmisYang LiJosipa BukićYang YangJosko BozicYang ZhangJoszef DudasYangpeiwei HuangJowy Yi Hoong SeahYan-Jye ShyongJózsef MihályYanka KaramalakovaJózsef PrechlYanling YanJózsef TollárYan-Ming XuJuan A. BernalYannis KaramanosJuan Bautista De SanctisYan-Ruide LiJuan Carlos Amor-DoradoYao-Chang ChiangJuan García-Bernalt DiegoYao-Ching HungJuan Jesús CruzYaojiong WuJuan Luis Garcia HernandezYaowu HeJuan Luis Muñoz BellidoYara M. MichelacciJuan M. Serrador PeiróYari LongobuccoJuan Maza-SolanoYaroslav A. AndreevJuan Mozas-MorenoYaroslav O. MezhuevJuan P. HenríquezYash GuptaJuan Pablo DamiánYasmyne C. RonquilloJuan PerdomoYasser HeakalJuan QinYasser RammahJuan R. De Los ToyosYasuhiko SugawaraJudit OláhYasuhiro KanataniJudith GreerYasuhiro OzekiJudy WongYasuhito IshigakiJuei-tang ChengYasuka MatsunagaJuilee RegeYasuko FujitaJui-Teng LinYasumasa IkedaJui-Yang LaiYasumasa OkazakiJulia CostaYasushi ShibataJulia EtichYasushige ShinguJulia GauerYasuyuki FujitaJulia SchuelerYasuyuki NagasawaJulia StadlerYegor E. YegorovJulian AnanievYehuda ShoenfeldJulian FriebelYen-Chien LeeJulian KenyonYen-Kung ChenJulian PohlanYen-Liang LiuJuliana FariaYen-Wen LiuJuliane Daßler-PlenkerYeongho KimJulianna KardosYeong-Min YooJulie Brogaard LarsenYet H. KhorJulie ChanYi HuangJulie FradetteYi Hui WuJulie RizzoYi Long TohJulien RossignolYi LuanJulien TailhadesYi WuJulio Cesar FranciscoYi XuJulio Plaza-DíazYiannis VasilopoulosJulita KulbackaYichen LiJun HarumaYi-Cheng HoJun KobayashiYi-Chou HouJun SawaiYifeng QiJun WanYi-Jang LeeJun WeiYi-Ju ChenJun-Bom ParkYijun PanJunchao TongYimin HuangJung Eun KimYiming ZhangJung ParkYin ZhaiJungwoo LeeYin-Ching ChanJunhua YangYing QingJunji YamauchiYinghuai ZhuJunli ZhaoYingjun LiuJunmei WangYing-Ming LiouJunmei ZhangYino WuJunrui ChengYiqiang ZhanJunsoo ParkYi-Ting HwangJürg HamacherYi-Yuan LinJürgen ArnholdYoana Rabanal RuizJürgen PannekYogesh SrivastavaJu-Seop KangYohan N’GuyenJustin MathesonYohei SekinoJustin S. AntonyYoichi EzuraJustyna E. GołȩbiewskaYolanda García-MesaJustyna GilYong ChenJustyna KowalskaYong DuJustyna MiszczykYong GeJustyna Opydo-SzymaczekYong Weon YiJustyna SikoraYong-Bin YanJu-Tae SohnYonggang ZhuJuvenal Rodriguez-ResendizYonghe MaJyh-fei LiaoYongji ZengK.S.V. Krishna RaoYongkang YangKaare GautvikYonglan LiuKabirullah LutfyYong-Moon LeeKah Kheng GohYoon-Seok ChungKah-Hui WongYoriyasu SuzukiKai QinYosep ChongKai TaoYoshi OdakaKai WangYoshiaki SunamiKai YuYoshiaki YuraKai ZhangYoshifumi IwagamiKai-Che WeiYoshihiro FukumotoKaihui LuYoshihiro IkuraKailash SinghYoshihiro ShidojiKai-Lee WangYoshikazu Honda-OkuboKai-Wen ChengYoshikazu UchidaKajal KhodamoradiYoshimasa AsoKalas WojciechYoshinobu KariyaKamalendra SinghYoshitaka NakayamaKamalika MojumdarYoshiya OdaKamil KrawczyńskiYoshiyuki NakamuraKamil KuderYoulim KimKamil NelkeYoung-Ji ShiaoKamila PolgarovaYoungsup SongKamila Środa-PomianekYousif SubhiKamilla GrzywaczYoussef RomanKamilla StachYousuf MohammedKamran IqbalYu KoyamaKamyar AsadipooyaYu SunKamyar ZahediYuan-Hung WangKarel AllegaertYub Raj NeupaneKarem A. CourtYu-Chan ChangKaren BohmwaldYue ShengKarin StrigårdYuen-Ki CheongKarina JouravlevaYuerou ZhangKarine MichaudYu-Hsiang KuanKarita PeltonenYu-Hsuan Joni ShaoKarl-Heinrich LinkYu-Huei LiuKarol SteckiewiczYuichi NishiokaKarolina GołąbekYuichiro J. SuzukiKarolina M. StepienYuji MurakamiKarolina Szewczyk-GolecYuji NadataniKároly GulyaYuji OeKarsten GroteYuji ShimizuKarthik H. ShankarYujiao LuKasandra BélangerYukihiko MomiyamaKatarina VukojevićYuko IwataKatarzyna BanachYulia SuzdaltsevaKatarzyna BergmannYulin RenKatarzyna Cieślik-BoczulaYuliya I. RaginoKatarzyna CzarzastaYumie TakataKatarzyna CzyżYun Hak KimKatarzyna D. SluzalskaYun LiuKatarzyna DziendzikowskaYun ShiKatarzyna HolcmanYunfeng LiuKatarzyna IżykowskaYunfeng ZhaoKatarzyna Kiec-KononowiczYung-Tsu ChoKatarzyna KorybalskaYunhai CuiKatarzyna KuterYun-Hee KimKatarzyna NabrdalikYuning ChenKatarzyna NeubauerYunsong LiuKatarzyna NowomiejskaYunwen TaoKatarzyna PalusYuqi ChenKatarzyna PankiewiczYuqing ZhangKatarzyna PierzchalaYuri KrakKatarzyna Sykłowska-BaranekYuriy I. GrinshteinKatarzyna Winsz-SzczotkaYuriy L. OrlovKatarzyna WyskidaYusuke IsshikiKatharina ProestlingYusuke OjiKatharina RichterYuta TanizakiKatharina RumpYutaka ItokazuKathleen HaleyYutaka KishidaKathryn FutregaYutaka OhsedoKatia BarbaroYutian ZouKatia LeiteYuwei FanKatia MesquitaYuxian HeKatia PaneYves St-PierreKatia StefanantoniZacharoula IatridiKatie DeCicco-SkinnerZachary F. BurtonKatja WitschasZachary T. GraberKatona GáborZackary FitzsimondsKatrina TraberZahra NasriKatsuhiko ArigaZaid YounesKatsuki TsuchiyamaZakariya AlgamalKatsuo KurabayashiZamzuri IdrisKatsura TakanoZanda BakaevaKavindra K. KesariZaneta Kimber-TrojnarKay-Dietrich WagnerZbârcea Cristina ElenaKazimierz KuliczkowskiZbigniew AdamiakKazuhiko KotaniZbigniew J. WitczakKazuhiro P. IzawaZbigniew Jan LeśnikowskiKazuki M. MatsudaZdenek KejikKazuki YasudaZehuan HuangKazumasa MatsumotoZehuan LiaoKazumoto MurataZekiye AltunKazunori KanemaruZelong XieKazunori NagasakaZhan ShuKazunori SangoZhaohai QinKazuto TajiriZhao-Qian TengKee Woei NgZhaowei ChenKee-Lung ChangZhaowei ZhangKei MaruyamaZhen LiKeigi FujiwaraZheng ZhongKeiko TanakaZhenghan WangKeisuke IshizawaZhengrong GuanKeitaro SouZheng-Yuan SuKeith RochfortZhenlin LiKeld-Erik BygZhenzhan WangKen MillsZhi Ping XuKen RosenthalZhibo MaKeng-Shiang HuangZhichao MaKenichi GotoZhidong ZhouKenichi TakayamaZhigao WangKenji SaitoZhimin ZhangKenneth Steven PlanteZhongbing LuKennio PaimZhongwei LiuKenry KenryZhubo WeiKentaro FukushimaZhuoxun WuKentaro YoshiokaZi-Bing JinKenza E. BenzeroualZihao OuKerstin MenckZijian ZhangKeshen LiZin Z. KhaingKeun-Yeong JeongZiqing ChenKevin A. FenixZiv RadisavljevicKevin ChenZivile UseckaiteKevin ItaŽivka DikaKevin MartellZixin ChenKevin WelchZlatko KopeckiKeyla Vargas-RománZofia Oko-SarnowskaKhairullo Faizullaevich MakhmudovZohreh IzadifarKhaled HamdenZoltán BánócziKhaled QanudZoltán GyöngyiKhosrow KashfiZong Sheng GuoKhrystyna O. SemenZoran MinicKi Hyun NamZorana MilanovicKi-Hong NamZoubir DjeradaKijin KimZrinjka Matek MišakKim TieuZrinka KovarikKim Van VlietZsolt DatkiKimberly E. StephensZsolt GállKimberly SullivanZsolt KőszegiKimberly TremblayZubair KhanKimmo VirtanevaZurab AzmaiparashviliKinga GawelZuzana AndrejčákováKinga GawlińskaZuzanna RzepkaKinga MusiałZvi BentwichKirat K. ChandZygmunt WarzechaKiyohide Usami



